# C-Abl is not actıvated in DNA damage-induced and Tap63-mediated oocyte apoptosıs in human ovary

**DOI:** 10.1038/s41419-018-1026-7

**Published:** 2018-09-20

**Authors:** Gamze Bildik, Ceyda Acılan, Gizem Nur Sahin, Sercin Karahuseyinoglu, Ozgur Oktem

**Affiliations:** 1 Graduate School of Health Sciences and School of Medicine, Koc University, Istanbul, Turkey; 2 Department of Molecular Biology and Genetics, School of Medicine, Koc University, Istanbul, Turkey; 3 Department of Histology and Embryology, School of Medicine, Koc University, Istanbul, Turkey; 4 Translational Research Laboratory in Reproduction and Cancer, Division Reproductive Endocrinology and Infertility, Department of Obstetrics and Gynecology, School of Medicine, Koc University, Istanbul, Turkey

## Abstract

There is a controversy in literature as to whether c-Abl is crucial for the induction of TAp63-mediated apoptosis and whether that inhibition of c-Abl with imatinib, which was designed to inhibit the oncogenic kinase BCR-ABL and c-kit, protects oocytes from chemotherapy-induced apoptosis in mice. No human data are available on this issue. We therefore aimed to explore whether genomic damage induced by chemotherapy drug cisplatin activates c-Abl along with TAp63 and the inhibition of c-Abl with imatinib prevents cisplatin-induced oocyte death and follicle loss in human ovary. Exposure to cisplatin induced DNA damage, activated TAp63 and SAPK/JNK pathway, and triggered apoptosis in the oocytes and granulosa cells. However, TAp63 activation after cisplatin was not associated with any increase in the expression of c-Abl. Imatinib did not prevent cisplatin-induced apoptosis of the granulosa cells or oocytes. Moreover, treatment with this drug resulted in the formation of bizarre shaped follicles lacking oocytes and increased follicular atresia by inducing apoptosis of granulosa cells and oocytes. Similar toxic effects were observed when ovarian tissue samples were incubated with a c-kit antagonist drug anti-CD117, but not with another c-Abl tyrosine kinase inhibitor GNF-2, which lacks an inhibitory action on c-kit. Intraperitoneal administration of imatinib to the xenografted animals produced similar histomorphological abnormalities in the follicles in human ovarian grafts and did not prevent cisplatin-induced follicle loss when co-administered with cisplatin. Our findings provide, for the first time, a molecular evidence for ovarian toxicity of this drug in human. Furthermore, this study together with two previous case reports of a severely compromised ovarian response to gonadotropin stimulation and premature ovarian failure in patients, while receiving imatinib, further heighten the concerns about its potential gonadotoxicity on human ovary and urge caution in its use in young female patients.

## Introduction

Cancer is one of the most important global public health problems all around the world. Thousands of young women are diagnosed with cancer every year and exposed to cytotoxic chemotherapy regimens and radiation, which have a substantial negative impact on reproduction^1^. They may cause infertility and premature ovarian failure by inducing genomic damage and apoptotic death of the oocytes and somatic cells in the ovary^2^. Therefore, preservation of gonadal function and fertility has become one of the major quality of life issues for cancer survivors at reproductive ages. Currently available fertility preservation strategies such as cryopreservation of gametes and ovarian tissue can help women achieve pregnancy and live birth after chemotherapy-induced premature ovarian failure. Nevertheless, these strategies cannot reverse menopause or restore ovarian function^3,4^. Therefore, any drug that preserves ovarian reserve by preventing follicle loss induced by cytotoxic chemotherapy drugs can potentially prolong reproductive life span and obviate the need for gamete freezing before cancer therapy. So far, different drugs and compounds were tested at in-vitro and in-vivo settings in animal studies to explore whether they protect oocytes from chemotherapy-induced apoptosis.

Imatinib mesylate is an inhibitor of the oncogenic tyrosine kinases c-Abl and c-kit used in the treatment of chronic leukemias and gastrointestinal tumors, respectively^5,6^. In 2009, Gonfloni et al.^7^ reported that c-Abl is activated along with TAp63 pathway upon exposure to chemotherapy agent cisplatin, and that inhibition of this tyrosine kinase with imatinib protects oocytes from cisplatin-induced death in mice. Three years later, another group of investigators obtained opposite results by showing that imatinib itself exerts a similar degree of cytotoxicity to cisplatin on the mouse ovaries and does not protect primordial follicle oocytes from cisplatin-induced apoptosis or prevent loss of fertility in two independent strains of mice^8^. The authors in the latter study also demonstrated that imatinib-sensitive kinases, such as c-Abl, are not required for the DNA damage-activated oocyte apoptosis that is mediated by TAp63. Furthermore, they raised their concerns about the gonadotoxic potential of this drug because of its inhibitory actions on c-kit, which is a survival factor for ovarian follicles^9^. In 2013, Kim et al.^10^ showed using in-vitro culture and subrenal capsule grafting of mouse ovaries that imatinib inhibits the cisplatin-induced apoptosis of oocytes within primordial follicles. In that study, the investigators demonstrated that cisplatin induces c-Abl and TAp73 expression in the oocyte. Although imatinib was unable to block cisplatin-induced DNA damage and damage response, such as the upregulation of p53, it inhibited the cisplatin-induced nuclear accumulation of c-Abl/TAp73, and the subsequent downregulation of TAp63 and upregulation of Bax, thereby abrogating oocyte cell death^10^. The same year Morgan et al.^11^ reported that imatinib protected follicles against damage induced by cisplatin but not doxorubicin in mouse newborn ovaries under in-vitro conditions. And very recently, Tuppi et al.^12^ demonstrated that TAp63 activation after exposure to chemotherapy drugs requires phosphorylation by both the priming kinase checkpoint kinase-2 (CHK-2) and the executioner kinase casein kinase-1 (CK1) in mouse primordial follicles, and that c-Abl is not involved in this process. Further, the inhibition of CK1 with a pharmacological inhibitor rescued primary oocytes from doxorubicin- and cisplatin-induced apoptosis^12^.

To date, no human data are available on this controversial issue. The effects of imatinib on human ovary is largely unknown, except for two separate case reports showing compromised ovarian function^13^ and premature ovarian failure in patients while on imatinib treatment^14^. We therefore aimed, in this translational research study, to determine ovarian effects of imatinib and explore whether genomic damage induced by chemotherapy drug cisplatin activates c-Abl along with TAp63 pathway and its inhibition with imatinib prevents apoptosis in human ovarian follicles, isolated oocytes, and granulosa cells using in-vitro experiments and in-vivo human ovarian xenograft model.

## Materials and methods

### Patients

Ovarian cortical tissues were obtained from patients (mean age ± SD: 27.2 ± 2.4, *n* = 20) undergoing laparoscopic excisions of benign ovarian cysts between the years 2015 and 2017. Discarded immature human oocytes at germinal vesicle (GV) (*n* = 40) and human luteal granulosa cells (HLGCs) were obtained during oocyte retrieval procedure from in-vitro fertilization (IVF) patients (*n* = 10). Informed consents were obtained from all patients and the study was approved by the Institutional Review Board (IRB) of Koc University (2015.204.IRB2.074).

### Human ovarian xenograft in nude mice

Human ovarian tissues obtained from the patients were minced under sterile conditions into small fragments of equal size (0.5 × 0.5 cm) and then xenografted subcutaneously to the right flank region of the 8-week-old nude mice (*n* = 4 per group). At 6 weeks post transplantation, the animals received a single intraperitoneal injection of cisplatin, imatinib, or cisplatin with imatinib. The drugs were used at the concentrations that were previously used in the papers of Gonfloni et al.^7^ and Kerr et al.^8^. Control animals received dimethyl sulfoxide (DMSO) only. The animals were killed and the xenografts were removed at 24 h. Intra-cardiac blood samples were obtained immediately post mortem for anti-Mullerian hormone (AMH) measurement.

### Human ovarian tissue culture

Ovarian cortices embedded in the cyst wall were removed under sterile conditions, minced into pieces of equal size (0.5 × 0.5 cm) and cultured for in 24-well format culture plate using 2 ml of culture media as we described previously^15^. The drugs were added to the culture media at the indicated concentrations.

### Culture medium formulation

HGrC1 and COV434 cell lines, HLGCs, and ovarian tissue samples were maintained at 37 °C with 5% CO_2,_ in Dulbecco’s modified Eagle’s medium/F12 supplemented with 10% (v/v) fetal bovine serum and 1% (v/v) Penicillin–Streptomycin Amphotericin B Solution. DMSO was used as vehicle drug.

### Oocyte culture

A total of 40 morphologically normal GV-stage oocytes were used in this study. They were incubated in the G-IVF culture medium (Vitrolife, Goteborg, Sweden). Imatinib and/or chemotherapy agents were added to the culture medium at the indicated concentrations.

### Human non-mitotic luteinized granulosa cells

HLGCs were recovered from follicular fluid during oocyte retrieval procedure in ten IVF patients. These cells are highly specialized primary luteinized granulosa cells. They do not proliferate either spontaneously; or after stimulation with a mitogenic agent. They produce large amounts of progesterone and estradiol hormones in vitro. The aspirates of follicular fluids were spun down at 500 g for 10 min. Then recovered cells were plated in 24-well format culture plate in a density of 5000 cells per well^15^.

### Mitotic non-luteinized human granulosa cell lines (HGrC1 and COV434)

This is a human non-luteinized mitotic granulosa cell line expressing enzymes related to steroidogenesis, such as steroidogenic acute regulatory protein, aromatase, and gonadotropin receptors^16^. COV434 cells were purchased from Sigma, Co. This cell line was generated from the cells obtained from granulosa cell tumor. The biological characteristics of this cell line include the production of 17 β-estradiol in response to follicle-stimulating hormone, the absence of LH receptor, no luteinization capability, and the presence of specific molecular markers of apoptosis enabling the induction of follicular atresia^17^.

### Chemicals and reagents

Imatinib mesylate and cisplatin were purchased from Cayman Chemicals (Cas# 220127-57-1, MI, USA) and Eli Lilly and Company (IN, USA), respectively. 4-hydroperoxy cyclophosphamide (4-OOH CY), the active in-vitro metabolite of the drug was purchased from Niomech (Bielefeld, Germany)^18^. Anti-CD117 neutralizing c-kit antibody and GNF-2, which is another BCR-ABL inhibitor without c-kit blocking actions, were obtained from Sigma-Aldrich Company GmbH (Germany).

All cell culture reagents, YO-PRO®−1 Iodide (491/509) and Alexa probes were purchased from Life Technologies (Thermo Fisher Scientific, Inc., MA, USA). Anti-cleaved caspase-3 (#9664), Anti-phospho-SAPK/JNK^Thr183/Tyr185^ (#9251), Anti-SAPK/JNK (#9252), Anti-phospho Chk1^Ser345^ (#2348), Anti-phospho Chk2^Thr68^ (#2197) antibodies, and Hoechst 33342 (#4082) were purchased from Cell Signaling Technology, Inc. (MA, USA). Anti-ƴH2A.X^Ser139^ antibody (clone JBW301) was from Millipore (MA, USA). Anti-TAp63 (EPR5701) and Anti-phospho-TAp63^Ser395^ antibodies were purchased from Abcam (USA). Anti-c-Abl (K-12, sc-131) and Anti-poly ADP ribose polymerase (PARP) (C2–10, 556362) antibodies were obtained from Santa Cruz Biotechnology, Inc. (CA, USA) and BD Biosciences (CA, USA), respectively. Anti-Vinculin antibody was from Sigma-Aldrich Chemie GmbH (Germany). xCELLigence system is a product of Roche Diagnostics (Mannheim, Germany). COV434 cell line was purchased from Sigma (St. Louis, MA, USA). HGrC1 was a gift from Dr Ikara Iwase (Nagoya University, Japan). All western blotting buffers and reagents were purchased from Bio-Rad.

### Histomorphometric assessment and follicle counts

Ovarian tissue samples were prepared for histomorphological assessment and follicle counts described previously^15^. Follicle density was determined from serial sections as the mean of follicle counts per square millimeter in every fifth section after staining (hematoxylin and eosin). Follicle density was expressed as follicle count/mm^2^. Light microscopic images were taken under a Zeiss Axioscope or Olympus IX71 (Japan).

### Immunoblotting

Proteins were separated by SDS-polyacrylamide gel electrophoresis and transferred onto a polyvinylidene difluoride membrane and then blocked with 5% non-fat dry milk in TBS-T (20 mM TrisHCl, pH 7.8, 150 mM NaCl, 0.1%, v/v Tween-20) at room temperature for 1 h. Then the primary antibodies at indicated dilutions were added and incubated rocking overnight at 4 °C. Anti-ƴH2AX and Cleaved Caspase-3 antibodies were used at 1:1000 and 1:500 dilutions to assess DNA damage and apoptosis, respectively. Anti-SAPK/JNK and Anti-Phospho-SAPK/JNK antibodies were both performed at 1:1000 dilutions. Anti-p63, Anti-Phospho-p63, and Anti-c-Abl antibodies were used at 1:500 dilutions. Anti-Vinculin at a dilution of 1:10,000 is used as a loading control. Secondary antibodies conjugated to horseradish peroxidase, anti-rabbit, and anti-mouse were used in 1:2000. Quantification of protein within membranes was done by using Clarity™ Western ECL Substrate. Chemiluminescence detections were performed by ChemiDoc XRS + Imaging System (Bio-Rad).

### Immunofluorescent staining

For immunofluorescence studies, treated cells were fixed with 10% neutral formalin for 20 min, washed twice with phosphate-buffered saline (PBS), and then treated with blocking buffer (1 × PBS/5% normal goat serum/0.3% Triton™ X-100) for 1 h. After rinsing with PBS, they were incubated overnight at 4 °C with cleaved caspase-3 antibody for detection of apoptosis and ƴH2AX antibody for detection of DNA damage by immunofluorescence. Primary antibodies were diluted in antibody dilution buffer (1 × PBS/1% bovine serum albumin/0.3% Triton™ X-100) at 1:50 and 1:200, respectively. After rinsing with PBS, the cells were incubated with fluorochrome-conjugated secondary antibody (Alexa 486, Molecular Probes, USA) diluted in antibody dilution buffer for 1 h. This step was followed by rinsing the coverslips slides and adding Hoechst 33342 (1 µg/mL) for DNA staining. The images were taken under appropriate channels using IF microscope (Olympus IX71).

### Real-time and quantitative assessment of cell proliferation and viability using xCELLigence system

The system uses specially designed microtiter plates containing interdigitated gold microelectrodes to non-invasively monitor the viability of cultured cells using electrical impedance as the readout and generates real-time curves of cell viability and proliferation. We previously used and described this system in our other studies that validated that this platform can be used as a tool to assess cytotoxic and mitogenic agents on human granulosa cells^15,19,20^. The electronic readout of cell-sensor impedance is displayed in real-time as cell index (CI), a value directly influenced by cell attachment, spreading, and/or cell proliferation. The cells were treated with the drugs at log phase. The viability and proliferation of the cells were monitored on the Real-Time Cell Analysis (RTCA) System at 30 min time intervals for up to 140 h. The results were expressed by normalized CI derived from the ratio of CIs before and after the addition of the compounds. The normalization of CI arbitrarily sets CI to 1 at the indicated time points. Recording of CI and normalization CI was performed using the xCELLigence RTCA Software 1.2.

### Cell viability assays

Cell viability was assessed using intravital carbocyanine YO-PRO-1 uptake assay or the cell titer-GLO® luminescent cell viability assay (CTG). YO-PRO-1 (0,1 µM) and Hoechst 33342 (1 µg/mL) were added to culture media at the indicated concentrations. Live/dead cell imaging of the cells were undertaken under appropriate channels using IF microscope (Olympus IX71) after 30 min of exposure. For CTG assay, the cells were plated at a density of 10,000 cells per well in a 96-well white, assay plate and treated with imatinib at the indicated concentrations. Cell Titer-Glo reagent was added at 1:1 volume ratio. The plate was placed on a rocking platform for 2 min and incubated at room temperature for 10 min before the luminescence signal was read in a plate reader (Thermo Scientific). Luminescence readings were background subtracted and normalized to control wells.

### Gene expression analysis via quantitative reverse-transcriptase PCR

RNA isolation was performed with Quick-RNA MicroPrep Kit (Zymo Research, Irvine, CA, USA) by following manufacturer’s instructions. RNA was quantified with spectrophotometric read at 260 nm by Nanodrop 2000 (Thermo Fisher Scientific, Inc.) and 1000 ng cDNA was prepared by using M-MLV Reverse Transcriptase (Invitrogen). Quantitative real-time expressions of mRNAs were detected and compared by using Light Cycler 480 SYBR Green I Master (Roche, Germany). The primers of the genes used in the study are shown below.

**Table:** 

*Gene*	*Sequence*	
CASP-3	F	5′-CATGGAAGCGAATCAATGGACT-3′
	R	5′-CTGTACCAGACCGAGATGTCA-3′
GAPDH	F	5′-AGCCACATCGCTCAGACAC-3′
	R	5′-GCCCAATACGACCAAATCC-3′
NOXA	F	5′-ACCAAGCCGGATTTGCGATT-3′
	R	5′-ACTTGCACTTGTTCCTCGTGG-3′
PUMA	F	5′-GACCTCAACGCACAGTACGAG-3′
	R	5′-AGGAGTCCCATGATGAGATTGT-3′

### Hormone assays

AMH levels in the supernatants were determined using Active Mullerian Inhibiting Substance/AMH (Diagnostic Systems Laboratories, Inc., USA) enzyme-linked immunosorbent kit. The analytical sensitivity of the kit was 0.006 ng/mL. Intra-assay repeatability and the coefficient of variations were given as 4.6% (0.144 ng/mL), 2.4% (0.843 ng/mL), and 3.3% (4.408 ng/mL), respectively. Estradiol level in the spent culture media was determined using the electro-chemiluminescence immunoassay, an immunoassay for the in-vitro quantitative determination of estradiol and (Elecsys® Estradiol II, Cobas) according to manufacturer’s instructions. All analyses were performed on Cobas® 6000 analyzer series (Roche Diagnostics, USA). Lower detection limits for estradiol was 18.4 pmol/L (5.00 pg/mL)

### Statistical analysis

Follicle counts, hormone levels, and cell index readouts of xCELLigence system were continuous data, therefore expressed as the mean ± [SD]. Analysis of variance and multiple comparison post-hoc test were used to compare continuous data among the groups if the data are parametric, or Kruskal–Wallis test and Dunn’s post-hoc comparison if it is non-parametric. Nominal data were compared between the groups using Fisher’ exact or *χ*^2^-tests where appropriate. Statistical analyses were done using SPSS for windows 21 statistical package program. *P* < 0.05 is considered significant.

## Results

### Human ovarian xenografts in nude mice

**Fig. 1:**
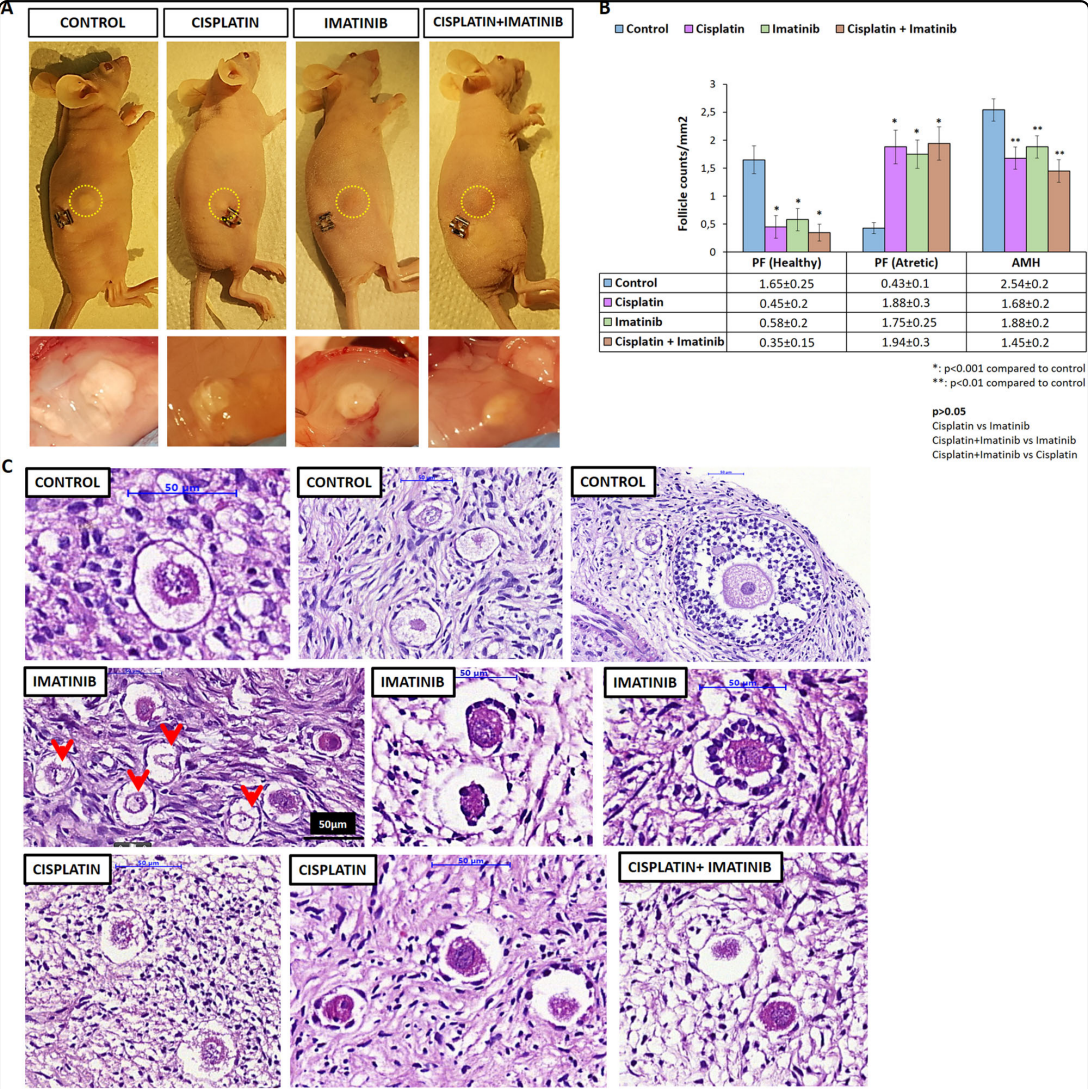
Human ovarian xenografting in nude mice. Intraperitoneal administration of a single dose of cisplatin 6 weeks after transplantation resulted in a drastic decrease in the number of primordial follicles along with a reciprocal increase in the number of atretic primordials in the xenografts at 24 h post exposure. The administration of imatinib 2 h before cisplatin did not prevent follicle loss. The reductions in the primordial follicle numbers were comparable among the animals treated with cisplatin, imatinib or both (**a**, **b**, and **c**). Primordials and small follicles lacking an oocyte and empty zona oocytes without granulosa cells were more abundantly observed in the xenografts exposed to imatinib (hematoxylin–eosin staining, red arrows **b**). Serum AMH levels were significantly decreased in the animals treated with cisplatin, imatinib, or both compared with control animals (**b**)

Treatment of the animals with a single intraperitoneal injection of cisplatin (5 mg/kg) 6 weeks after xenografting of human ovarian samples resulted in a significant decrease in the number of healthy primordial follicles in xenografted human ovarian tissues along with a reciprocal increase in the number of atretic primordial follicles at 24 h post injection compared with control animals receiving vehicle drug DMSO injections. The administration of imatinib (7.5 mg/kg) before (2 h) cisplatin did not appear to confer any protective effect against cisplatin-induced follicle loss, because the number of primordial follicles were comparable between the xenografts exposed to cisplatin vs. cisplatin + imatinib (Fig. 1a, b). Interestingly, the magnitude of the gonadotoxicity after imatinib appeared to be similar to cisplatin when a comparison was made based on the degree of follicle loss and the reduction in AMH levels. Notably, bizarre shaped primordial follicles lacking oocytes and unclassifiable small follicles possessing atretic oocytes without granulosa cells were much more frequently observed in the samples exposed to imatinib in comparison with control xenografts and those exposed to cisplatin (33% vs. 1% vs. 8% *P* < 0.01, respectively) (Fig. 1c). We were not able to assess the effect of cisplatin and imatinib on the secondary follicles (namely, primary, pre-antral and antral stage follicles) as they constitute approximately 10% of total follicle pool in human ovary, precluding us from making a quantitative analysis.

Histological examination of the animals’ own ovaries revealed similar findings to what we obtained in the ovarian xenografts. The number of primordial follicles was significantly reduced in the mouse ovaries treated with cisplatin. Imatinib treatment with cisplatin did not prevent cisplatin-induced follicle loss. Furthermore, primordial follicle number was substantially reduced and there were multiple small follicles lacking oocytes in the animals receiving imatinib alone without cisplatin. On the other hand, the number of pre-antral and antral follicles were comparable among the control animals and those treated with cisplatin, imatinib, or both (Supplementary Figure 1).

These in-vivo findings provided a histomorphological evidence for possible gonadotoxic effects of imatinib on human ovary and prompted us to analyze ovarian effects of this drug more thoroughly using the following ex vivo and in-vitro experiments.

### Ex vivo treatment of ovarian cortical samples with cisplatin, imatinib, or both

**Fig. 2:**
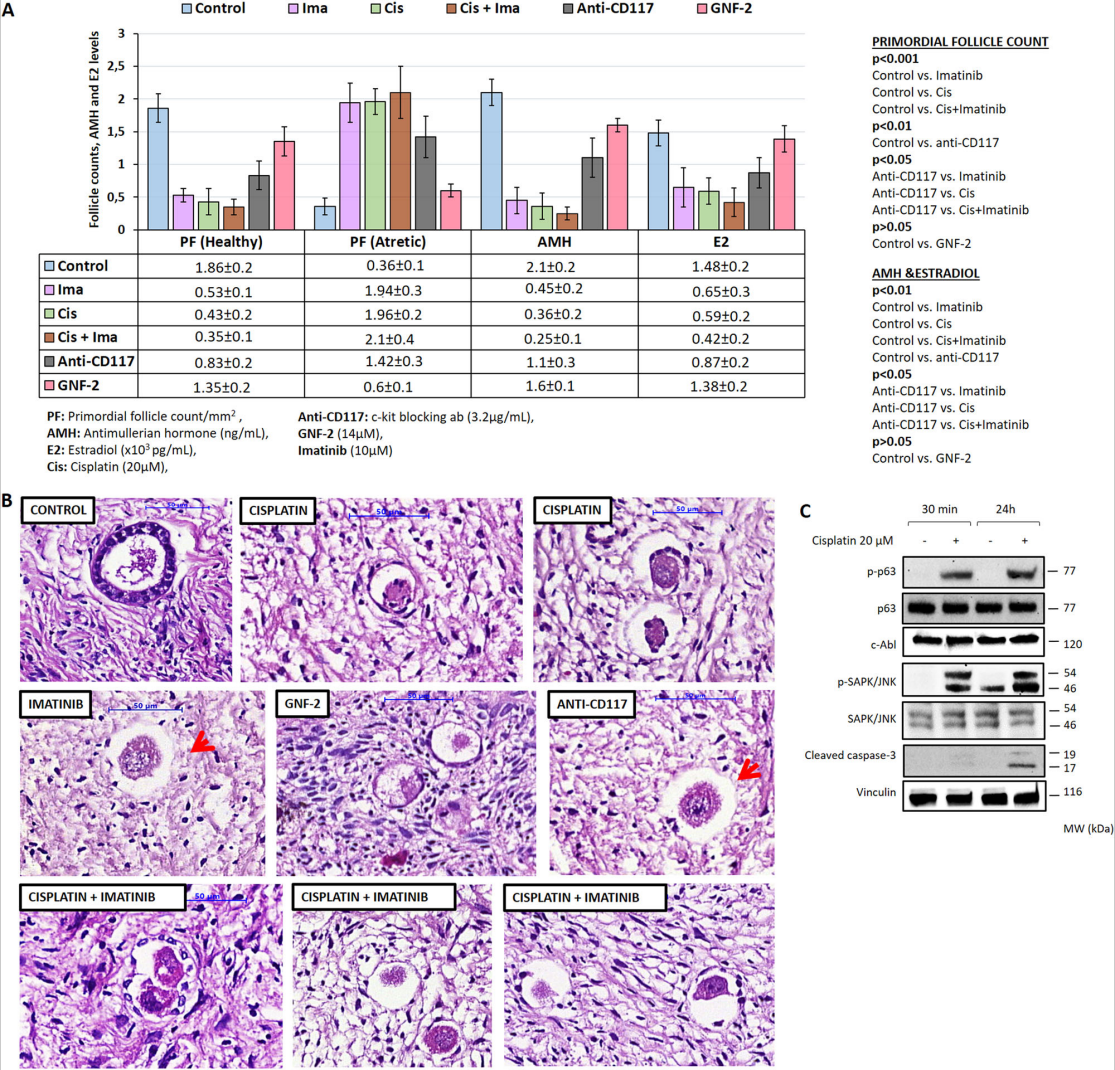
Ovarian tissue culture model. In-vitro treatment of ovarian cortical pieces with cisplatin for 24 h resulted in significant reductions in the number of primordial follicles and in-vitro estradiol (E_2_) and AMH production of the samples along with an increase in the number of atretic follicles. Imatinib treatment 2 h before cisplatin neither ameliorated cisplatin-induced follicle loss, nor improved in-vitro hormone productions of the samples compared to those treated with cisplatin alone (**a**). The reduction in follicle counts and hormone productions were comparable among the samples exposed to cisplatin, imatinib, or both. Follicle atresia at such a degree was also observed in the samples exposed to c-kit blocking agent anti-CD177 but not in those treated with another c-Abl inhibitor GNF-3, which does not possess c-kit blocking action (**a** and **b**). Bizarre shaped follicles and small follicles lacking an oocyte were more commonly observed in the samples treated with imatinib and anti-CD177 but not in the control and cisplatin-treated ones (red arrows, **b**). Exposure of human ovarian tissue to cisplatin at 20 µM concentration for short (30 min) and long (24 h) terms induced SAPK/JNK and TAp63 pathways, and triggered apoptosis as evidenced by an increase in the protein expression of cleaved form of caspase-3 in immunoblotting. However, TAp63 activation after cisplatin was not associated with any notable change in the protein level of c-Abl (**c**)

Treatment of ovarian cortical pieces in culture with cisplatin for 24 h resulted in significant reductions in the number of healthy primordial follicles and in-vitro estradiol (E_2_) and AMH production of the samples along with a reciprocal increase in the number of atretic follicles. Imatinib treatment before or concurrent with cisplatin neither ameliorated cisplatin-induced follicle loss nor improved in-vitro hormone productions of the samples compared with those treated with cisplatin alone (Fig. 2a). When a comparison was made based on the healthy and atretic fractions of the primordial follicles, and E_2_ and AMH levels, imatinib and cisplatin appeared to exert a similar degree of cytotoxicity on the ovarian tissue samples under in-vitro conditions. Furthermore, in-vitro imatinib treatment resulted in the morphological abnormalities in the follicles in the ovarian tissue samples that were similar to those observed in the ovarian xenografts of the animals treated with imatinib. Namely, bizarre shaped primordial follicles, follicles lacking granulosa cell layer, and/or oocytes and empty zona oocytes were more commonly observed in the samples treated with imatinib compared to control and cisplatin-treated ones (42% vs. 2% vs. 7%, *P* < 0.01) (Fig. 2b). In another set of experiments, we repeated ovarian tissue culture experiments with imatinib used at three different concentrations and found that follicle atresia was increased and steroidogenic activity of the samples was decreased with increasing the concentration of the drug, suggesting that ovarian toxicity of imatinib is dose dependent (Supplementary Figure 2).

**Fig. 3:**
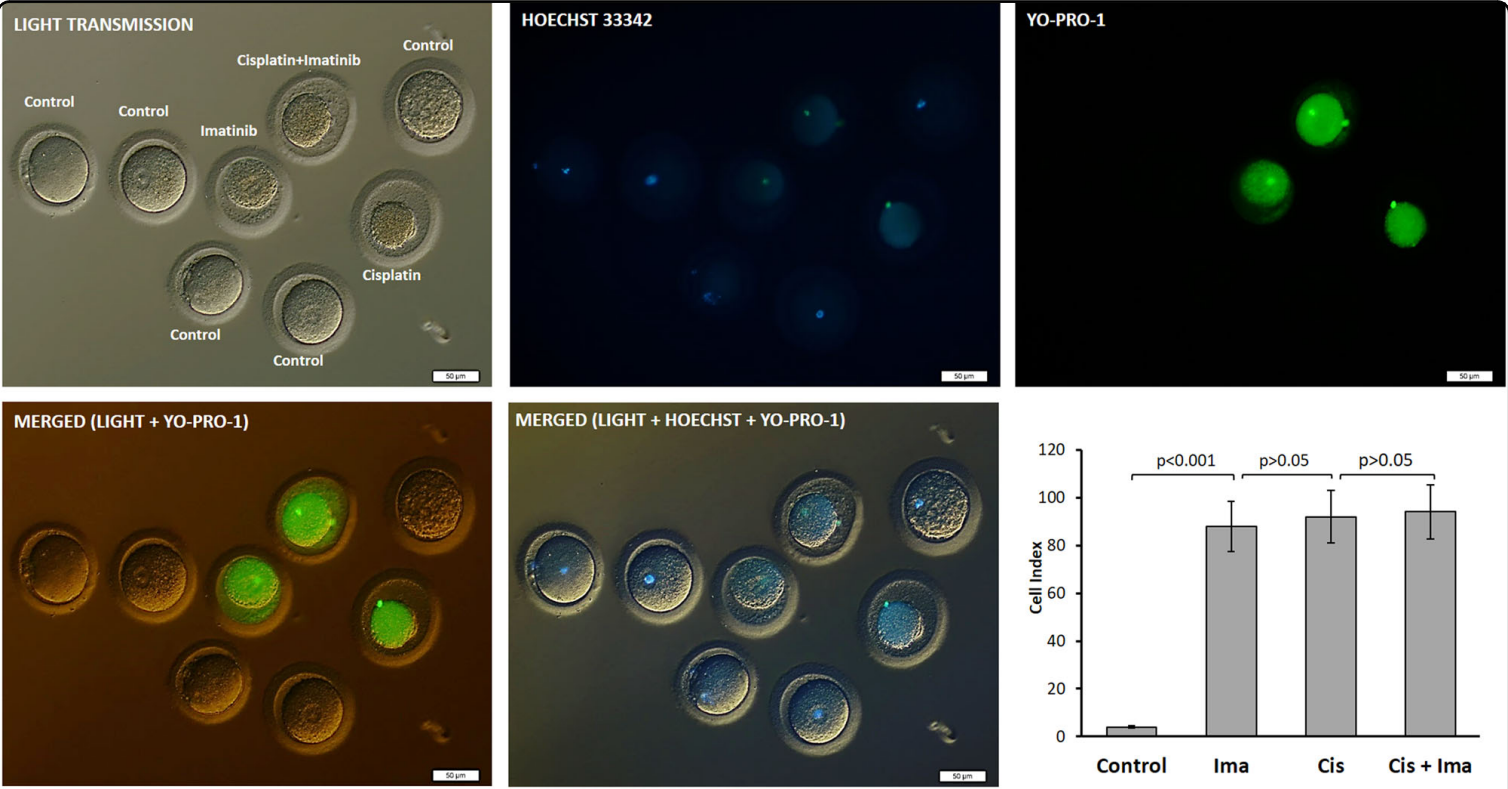
In-vitro exposure of isolated oocytes to cisplatin, imatinib, or both. Although only 2% of the control oocytes underwent atresia after 12 h incubation period, the rate of apoptosis was significantly increased to 82%, 88%, and 92% after treatment with imatinib, cisplatin, and cisplatin with imatinib, respectively (*P* < 0.001). Imatinib treatment before with cisplatin did not rescue the oocytes from atresia

Abundance of small follicles lacking oocytes in the ovarian samples exposed to imatinib led us to investigate the effect of this drug on individual oocytes obtained from IVF patients. We observed that exposure of the oocytes to imatinib for 12 h tremendously increased the rate of atresia to 82%. During the same incubation period, only 2% of the control oocytes exposed to DMSO as vehicle drug underwent atresia (*P* < 0.001). Cisplatin exposure caused a similar degree of oocyte atresia (88%) and imatinib treatment 2 h before cisplatin did not rescue the oocytes from apoptosis (92%, *P* > 0.05) (Fig. 3).

Based on the in-vivo and in-vitro findings that we obtained so far, we hypothesized that the observed toxic effects of imatinib on human ovary might be related to its inhibitory actions on c-kit, which is another tyrosine kinase crucial for survival of ovarian follicles^21^. For this purpose, we repeated ovarian tissue culture experiments with GNF-2, another c-Abl tyrosine kinase inhibitor lacking c-kit blocking action, and with anti-CD117, which is a pure c-kit blocking drug. We found that although there were no significant differences between control and GNF-2-treated samples in terms of follicle counts and in-vitro E_2_ and AMH productions, treatment with anti-CD117 was associated with significant reduction in the primordial follicle count and in-vitro hormone productions of the samples compared with control and GNF-2-treated pieces (Fig. 2a). When a comparison was made between the ovarian samples exposed with imatinib vs. anti-CD117, it appeared that imatinib was associated with a greater reduction in primordial follicle number, E_2_, and AMH levels, suggesting that the detrimental effects of imatinib on the follicles includes but is not limited to its inhibitory actions on c-kit. These results also show that anti-CD117 had a moderate and GNF-2 had the least ovarian toxicity. It is also worthwhile to note that bizarre shaped primordial and unclassifiable small follicles possessing atretic oocytes without granulosa cells were much more abundantly observed in the samples exposed to imatinib (45% of the atretic follicles) and anti-CD177 (37%) compared with those treated with GNF-2 (2%) or cisplatin (6.3%) (Fig. 2b).

### TAp63 activation by cisplatin-induced genomic damage is not associated with upregulation of c-Abl expression in ovarian cortical samples, isolated oocytes, and granulosa cells

**Fig. 4:**
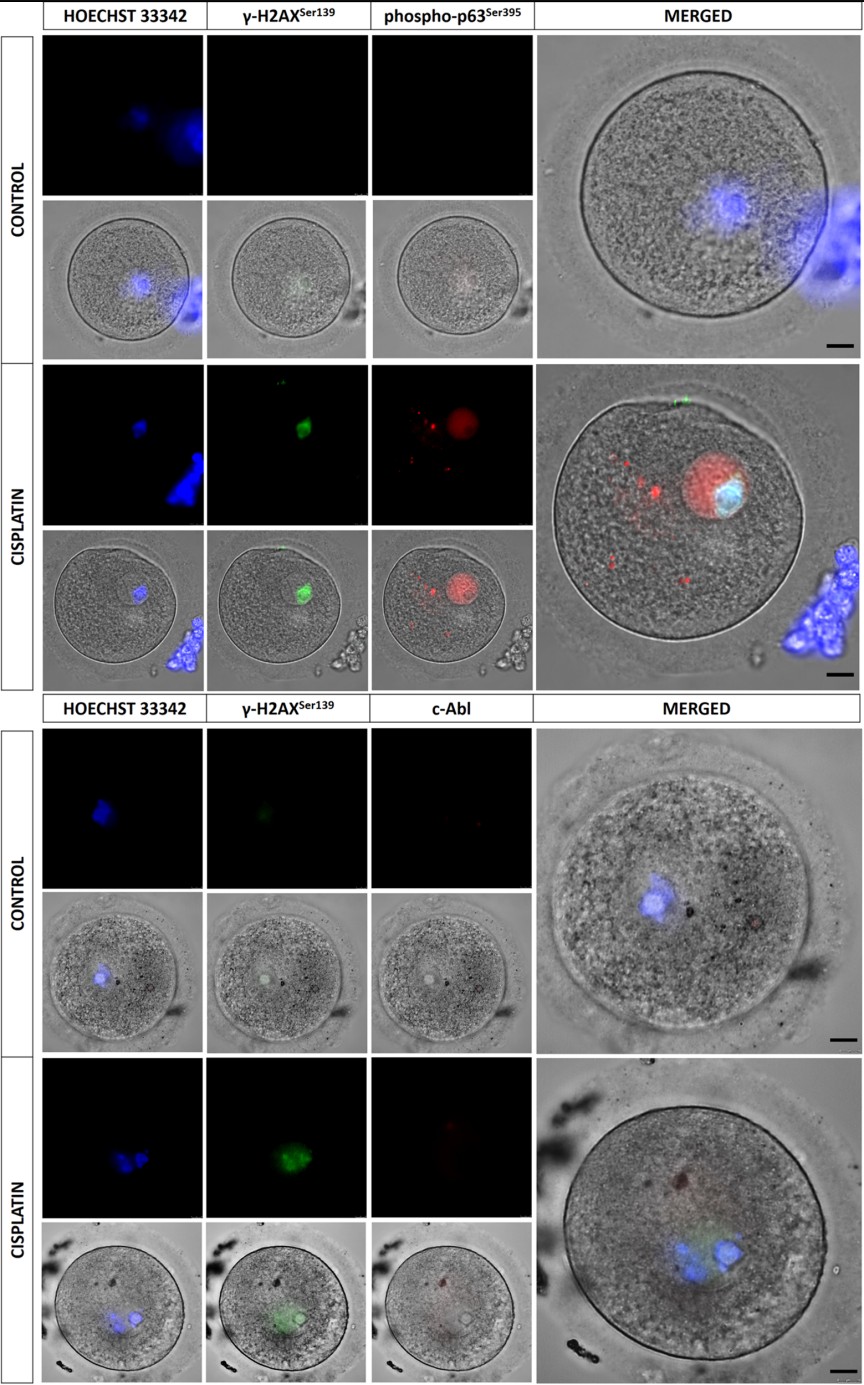
The expression of TAp63 and c-Abl in human oocytes before and after exposure to cisplatin in vitro. Exposure of the oocytes to cisplatin (20 µM) for 5 min increased the expression of γ-H2AX phosphorylation ^Ser139^ and phospho-TAp63^Ser395^ compared with control oocytes. However, there was no signal of c-Abl detected in the oocytes after cisplatin exposure despite the marked expression of γ-H2AX phosphorylation ^Ser139^ as an evidence for the occurrence of double-strand DNA breaks after cisplatin

In order to address the question as to whether genomic damage induced by cisplatin activates c-Abl along with TAp63 pathway in human ovary, we utilized ovarian cortical tissue samples, isolated oocytes and three different type of granulosa cells. Exposure of the tissue samples to cisplatin at short (30 min) and longer durations (24 h) activated SAPK/JNK and TAp63 pathways, and triggered apoptosis as evidenced by increased expressions of the phosphorylated forms of SAPK/JNK^Thr183/Tyr185^, p63^Ser395^, and cleaved form of caspase-3 on immunoblotting. However, TAp63 activation after cisplatin exposure was not associated with any notable change in the protein expression of c-Abl (Fig. 2c). Similarly, incubation of the oocytes with cisplatin for 5 min induced double-strand DNA damage and activated TAp63 as shown by increased expressions of γ-H2AX phosphorylated at Ser139 and phospho-p63 ^Ser395^ on immunofluorescence staining. However, there was no signal of c-Abl detected before or after cisplatin treatment (Fig. 4).

**Fig. 5:**
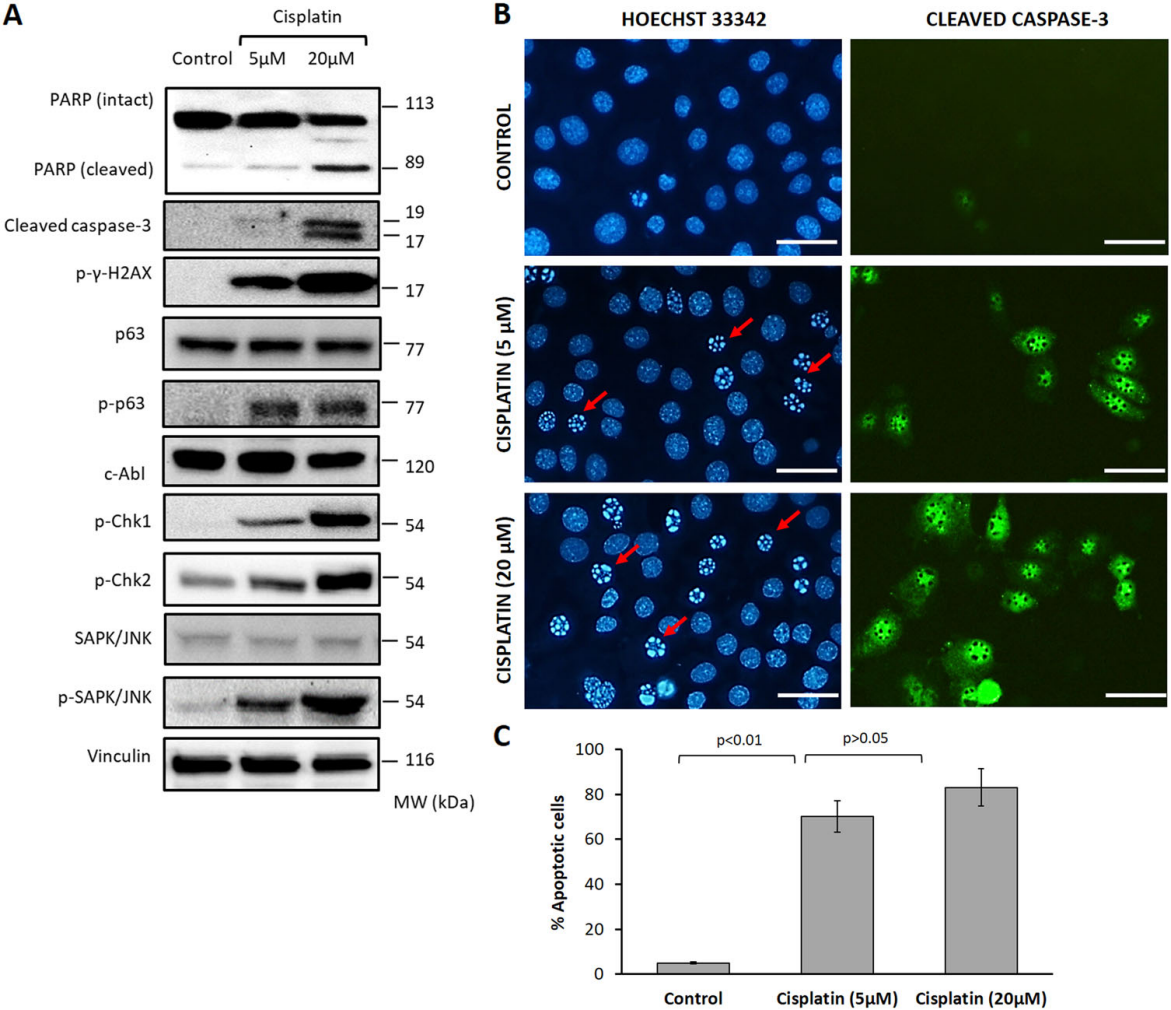
In-vitro exposure of human granulosa cells to cisplatin. Exposure of HGrC1 cells to cisplatin at 5 and 20 µM concentrations for 4 h induced genomic damage, activated cell cycle checkpoint sensors, SAPK/JNK and TAp63 pathways, and triggered apoptosis as evidenced by a dose-dependent increase in the protein expression of the γ-H2AX phosphorylation ^Ser139^, phosphorylated forms of Chk-1 and Chk-2, SAPK/JNK^Thr183/Tyr185^, p63^Ser395^, and cleaved forms of PARP and caspase-3, respectively, in immunoblotting. However, TAp63 activation after cisplatin was not associated with any notable change in the protein level of c-Abl (**a**). Apoptotic signal and nuclear fragmentation became evident at 24 h post exposure in immunofluorescence analysis confirming apoptosis-inducing effects of cisplatin at these concentrations (**b** and **c**)

*γ*-H2AX ^Ser139^ foci appeared as early as 5 min post exposure after cisplatin treatment of HGrC1 granulosa cells and became more pronounced at 30 min, 2 h, and 4 h after exposure in immunofluorescence analysis (Supplementary Figure 3). In immunoblot analysis, exposure of HGrC1 cells to cisplatin at two different concentrations for 4 h induced genomic damage, activated cell cycle checkpoint sensors, SAPK/JNK and TAp63 pathways, and triggered apoptosis as evidenced by a dose-dependent increase in the expression of the phosphorylated forms of *γ*-H2AX ^Ser139^, Chk-1^Ser345^ and Chk-2^Thr68^, SAPK/JNK^Thr183/Tyr185^, p63^Ser395^, and cleaved forms of PARP and caspase-3. However, TAp63 activation after cisplatin was not associated with any increase in the protein level of c-Abl (Fig. 5a). At 24 h post exposure, nuclear fragmentation and apoptosis of the cells became evident in immunofluorescence analysis (Fig. 5b, c).

**Fig. 6:**
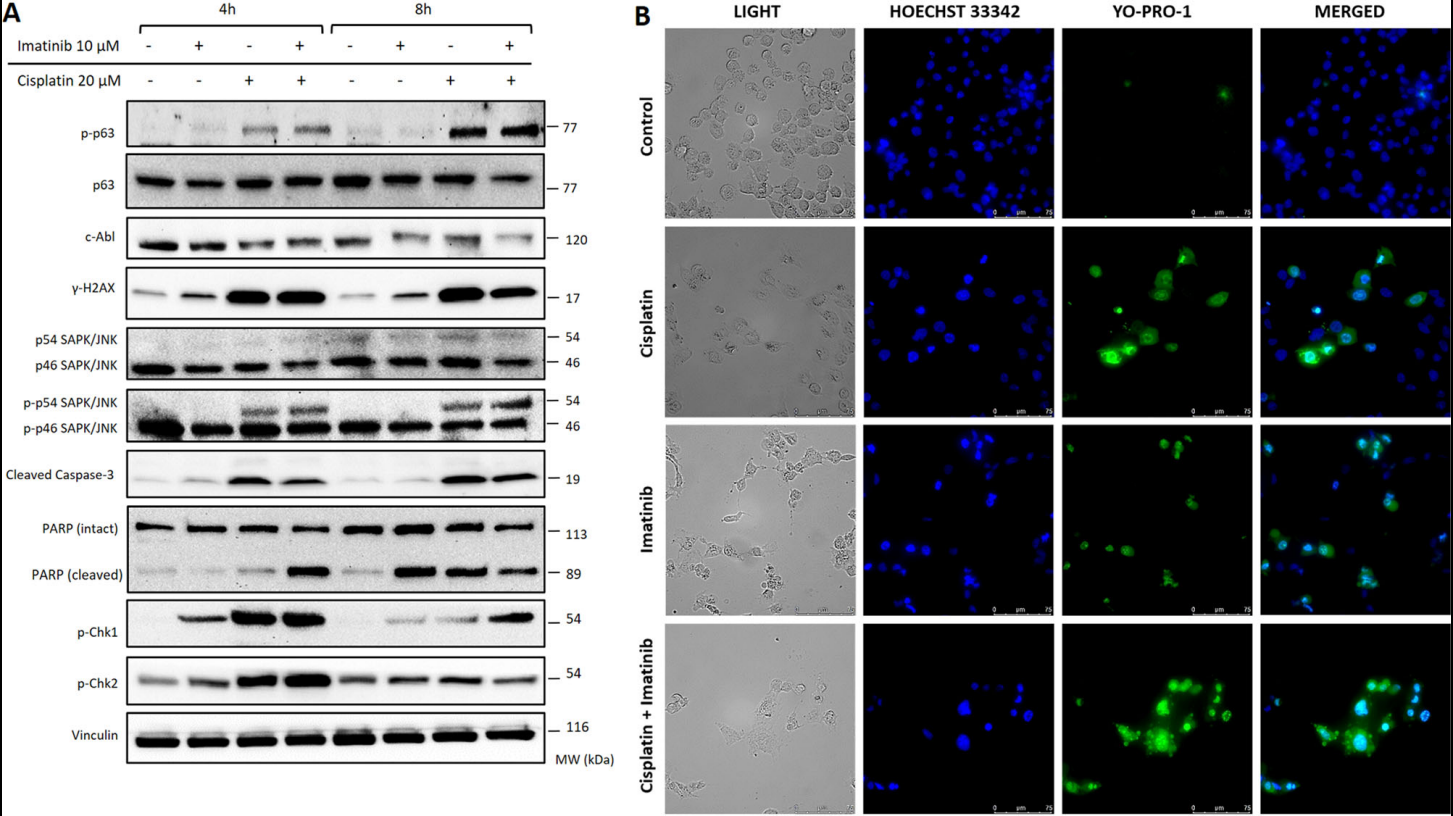
Analysis of the expression of the markers of genomic damage, cell cycle checkpoint, apoptosis, and viability of granulosa cells (HGrC1) exposed to cisplatin, imatinib, or both. Administration of imatinib 2 h before cisplatin did not cause any notable change in the expression of c-Abl and phosphorylated forms of у-H2AX, Chk-1/Chk-1, SAPK/JNK, and p63 compared with those treated with cisplatin alone in immunoblotting analysis at 4 and 8 h post exposure (**a**). When cell viability was assessed with intra-vital YO-PRO-1 on immunofluorescence, it appeared that cisplatin treatment resulted in a significant reduction in the number of viable cells compared with control samples. Imatinib treatment did not prevent cisplatin-induced death of the cells (**b**)

In human granulosa cell line HGrC1, administration of imatinib alone or 2 h before cisplatin slightly decreased the expression of c-Abl, but did not cause any notable decrease in the expression of the phosphorylated forms of у-H2AX, Chk-1/Chk-1, SAPK/JNK, and TAp63 compared with those treated with cisplatin alone in immunoblotting analysis (Fig. 6a), nor it rescued the cells from apoptosis induced by cisplatin (Fig. 6b). Similar results were obtained in other two types of granulosa cells and when the experiments were repeated with another chemotherapy drug of 4-OOH CY, in-vitro active metabolite of cyclophosphamide, suggesting that the absence of c-Abl upregulation after TAp63 activation after genomic damage is not specific to a particular type of granulosa cells or chemotherapy drug (Supplementary Figures 4 and 5).

We also carried out an reverse-transcriptase PCR profiler assay to see the mRNA expression of NOXA and PUMA before and after cisplatin, as these pro-apoptotic proteins function as downstream transcriptional targets of TAp63 to mediate primordial follicle oocyte apoptosis at least in mice^22^. Although there is an almost twofold increase in NOXA expression in the ovarian cortical samples, HGrC1 and COV434 cell lines exposed to cisplatin for 24 h PUMA expression did not change. By contrast, in the luteal granulosa cells, PUMA was increased by 4.5-fold but NOXA was significantly downregulated after the same duration of exposure to cisplatin, suggesting that granulosa cell apoptosis upon exposure to cisplatin downstream TAp63 is differentially regulated depending upon the type of granulosa cells in human (Supplementary Figure 6).

**Fig. 7:**
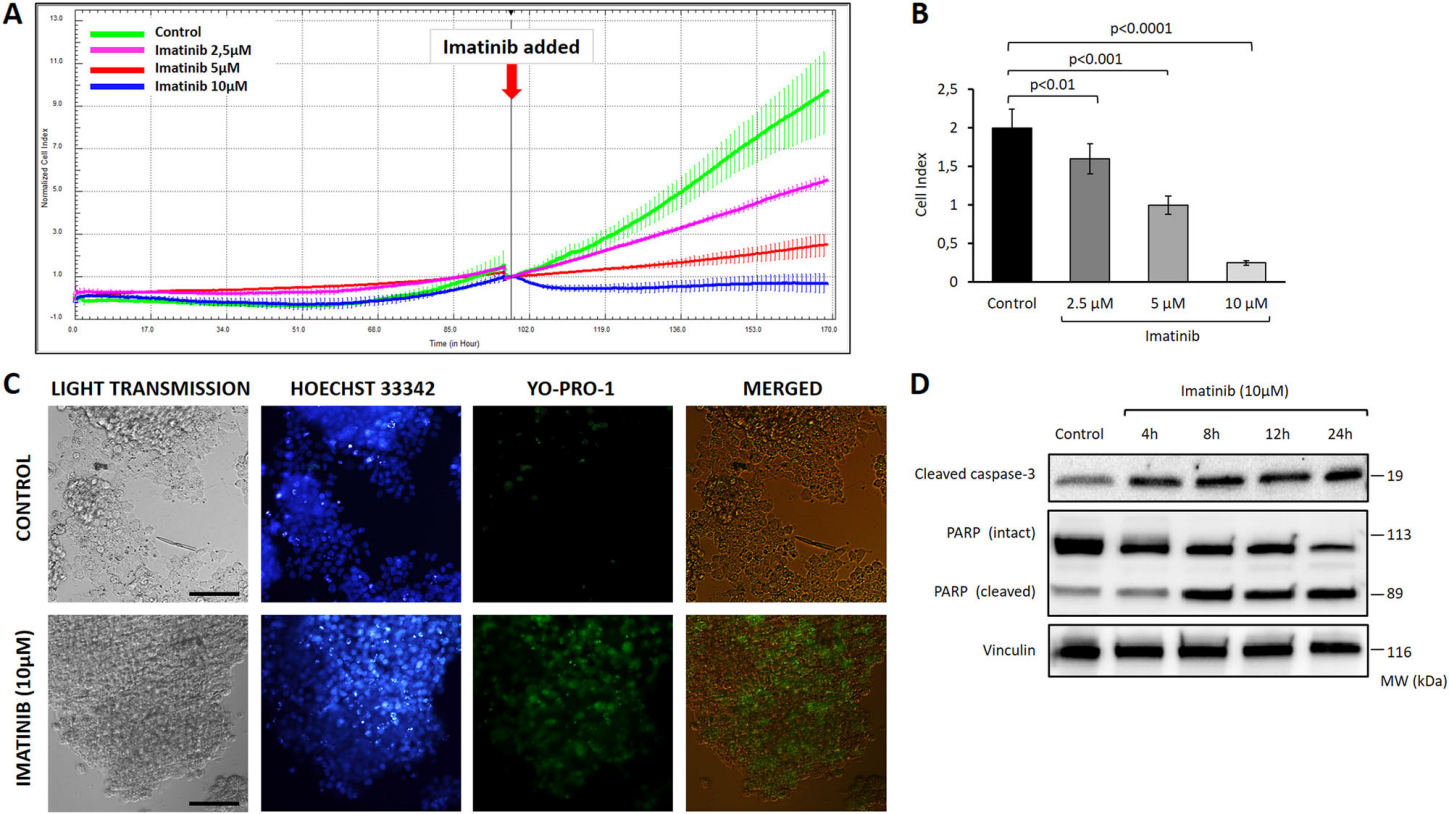
Assessment of the effects of imatinib on the proliferation and viability of human granulosa cells (HGrC1). Treatment of the cells with imatinib at the indicated concentrations caused dose-dependent growth arrest in the real-time growth curves of the cells on the xCELLigence system (**a**). The mean cell index as measure of viable cell mass declined with increasing concentrations of imatinib (**b**). Cell death was confirmed with intravital YO-PRO1 staining in immunofluorescence microscopy (**c**) and by increased expression of cleaved forms of caspase-3 and PARP on immunoblotting (**d**). There were dose and exposure time-dependent decrease in the proliferation and increase in the apoptosis of the cells when they were treated with imatinib

We also observed that imatinib treatment alone increased the expression of у-H2AX^Ser139^ in the granulosa cells at 4 and 8 h after exposure in immunoblotting (Fig. 6a). This effect of imatinib was shown previously on the different types of malignant cells on culture including leukemia and gastrointestinal stromal tumor cells in the prediction of response to therapy^23,24^. Therefore, in another set of experiment the granulosa cells were treated with imatinib at different concentrations and extended culture period up to 24 h to analyze long-term effect of imatinib exposure on granulosa cell viability and proliferation. Imatinib treatment caused dose-dependent growth arrest and induced apoptosis of the mitotic granulosa cell line HGrC1 (Fig. 7a, b). Decreased viability and apoptosis of the cells after exposure to imatinib was also confirmed with YO-PRO-1 vitality assay on immunofluorescence staining and increased expression of cleaved forms of caspase-3 and PARP on immunoblotting analysis (Fig. 7c, d). We also conducted a conventional CTG cytotoxicity assay on the mitotic HGrC1 and COV434 granulosa cells and obtained similar findings (Supplementary Figure 7).

**Fig. 8:**
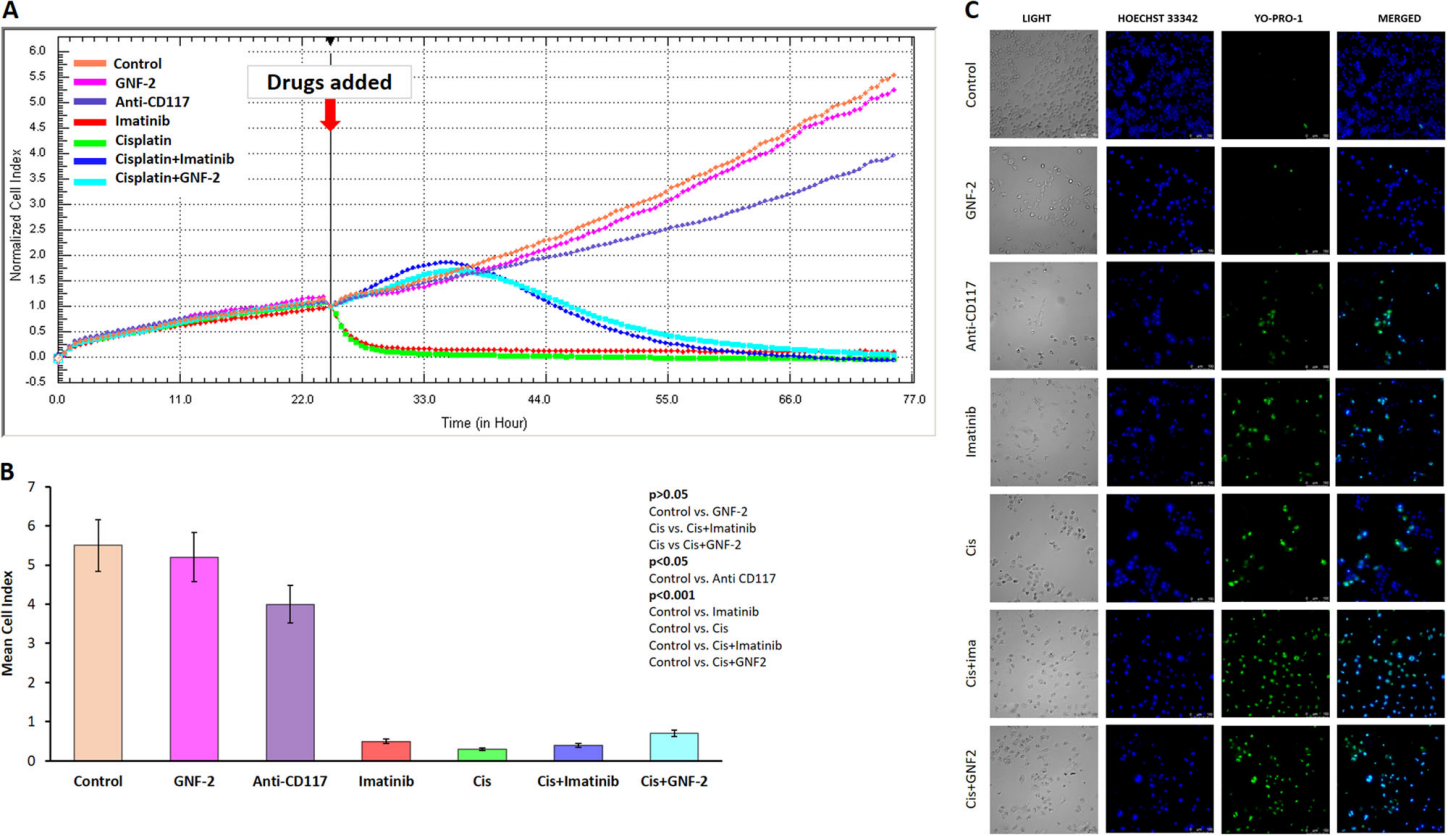
Real-time growth curves and viability of the cells treated with imatinib, GNF-2, anti-CD117, and cisplatin in vitro. The greatest reduction in proliferation and viability was observed in the cells exposed to cisplatin followed by in order of decreasing cytotoxicity imatinib and anti-CDD17 and GNF-2. Indeed, the proliferation rate of the cells treated with GNF-2 was comparable to control. The co-administration of imatinib or GNF-2 with cisplatin did not rescue the cell from apoptosis (**a**, **b**, and **c**)

The expression of c-Abl was gradually diminished on immunoblotting when the HGrC1 granulosa cells were treated with increasing concentrations of imatinib and GNF-2, confirming in-vitro functionality of these drugs (Supplementary Figure 8). As the last part of the experiments, we analyzed how mitotic activity and viability of the granulosa cells change when they were exposed to imatinib, GNF-2, anti-CD177, and cisplatin with and without imatinib or GNF-2 by monitoring real-time growth curves of the cells on the xCELLigence platform and assessing their viability on immunofluorescence analysis. The greatest reduction in proliferation and viability was observed in the cells exposed to cisplatin followed by in order of decreasing cytotoxicity imatinib, and anti-CDD17 and GNF-2. Indeed, the proliferation rate of the cells treated with GNF-2 was comparable to control. The co-administration of imatinib or GNF-2 with cisplatin did not rescue the cell from apoptosis (Fig. 8).

## Discussion

Infertility and premature ovarian failure are reproductive sequels of exposure to cytotoxic chemotherapy regimens in young females with cancer. They cause follicle death by inducing genomic damage in the oocyte and somatic cells of dormant primordials and growing follicles^2,25,26^. Premature ovarian failure is also associated with other adverse health-related consequences, including osteoporosis, hot flashes, sleep disturbance, and sexual dysfunction, which can negatively impact on short- and long-term quality of life. Ovarian tissue banking cannot fully restore ovarian function after transplantation and reverse menopause^3,4^. Furthermore, this strategy is considered still experimental and therefore should be performed under IRB approval and guidance^27^. Even though oocyte or embryo freezing before chemotherapy can help women achieve pregnancy and live birth after chemotherapy-induced premature ovarian failure, these strategies cannot reverse menopause. Therefore, focusing on research to discover new pharmacological options to protect ovarian follicles against chemotherapy-induced death in the ovary should be a priority. Obviously, such an achievement may prevent premature menopause and prolong reproductive life span by preventing total exhaustion of follicle stockpile in the ovary exposed to gonadotoxic cancer drugs.

So far, several pharmacological agents and compounds were tested at in-vitro and in-vivo animal studies to explore their protective effects against chemotherapy-induced follicle damage/loss in the ovary. Imatinib is such a drug that inhibits oncogenic tyrosine kinases c-Abl and c-kit used in the treatment of chronic leukemias and gastrointestinal stromal tumors respectively^5,6^. As the initial report that showed the protective effect of this drug on chemotherapy-induced follicle loss in mouse ovary several other studies were conducted with inconsistent results^7–12^. Lack of human data on this controversial issue became our motivation in conducting this translational study and we have obtained several important findings as follows. First, genomic damage induced by chemotherapy drug cisplatin is associated with increased expression of TAp63 phosphorylated at Ser395 and Ser160/162 residues, but does not require c-Abl upregulation in human ovarian cortical samples, isolated oocytes and granulosa cells. To the best of our knowledge it is a novel finding in human ovary, as no previous studies documented such an association between TAp63 phosphorylation at these residues and DNA damage induced by chemotherapy exposure. Second, imatinib mesylate did not confer any protection against cisplatin-induced follicle loss in human ovary samples, different types of granulosa cells, and oocytes in in-vitro experiments and in-vivo human ovarian xenograft model. Third, imatinib itself exerted cytotoxic effects on human ovary based on the reduction of follicle pool and steroidogenic activity of ovarian samples. Forth, there were specific structural abnormalities in the follicles that were much more frequently observed in the ovarian samples exposed to imatinib and c-kit blocking drug anti-CD117 but not in those incubated with cisplatin or GNF-2, another c-Abl inhibitor devoid of c-kit blocking action, suggesting that ovarian toxicity of imatinib is mainly related to its inhibitory actions on c-kit. These results support the findings of Kerr et al.^8^ in the mouse ovary and provide for the first time a molecular evidence in human that imatinib might be gonadotoxic effects on human ovary and does not confer any protection against cisplatin-induced follicle death in human. In addition, our findings together with two separate cases of a severely compromised ovarian response to gonadotropin stimulation and premature ovarian failure in patients who were receiving imatinib^13,14^ further heighten the concerns about its potential gonadotoxicity on human ovary and urge caution in its use in young female patients.

TAp63a is a member of the Trp53 family (Trp53, Trp63, and Trp73), which is predominantly expressed by the oocytes of the primordial follicles^28^ and essential for oocyte death after genotoxic stress^29^. As an oocyte-specific homolog of p53 TAp63 is expressed in meiotically arrested oocytes and act as a post-pachytene checkpoint to eliminate the oocytes, which have sustained DNA damage^12^. It is phosphorylated upon induction of double strand breaks in the DNA, which triggers conformational change and results in the formation of an open active and tetrameric structure^30^. As transcription targets of TAp63 pro-apoptotic BH3 only proteins PUMA and NOXA coordinate DNA damage-induced, TAp63-mediated primordial follicle oocyte apoptosis in mice^22^. These data were obtained in mouse oocytes and no data is available in human oocytes. Therefore, it is unclear if there is a conformational change in TAp63 upon induction of DNA damage in human oocyte. Unfortunately we did not answer this question in the current study, which will be the subject of our next study.

In human fetal ovary, no staining for p63 was observed in the oogonia and during early prophase-I, using ovaries obtained at between 7 and 15 weeks of gestation. Strong staining of late pachytene- and diplotene-stage oocytes was detected in ovaries obtained at 24 and 26 weeks of gestation^28^. No data is available regarding its expression pattern in the adult human ovary. In this study, we have shown that TAp63 is expressed not only by oocytes but also granulosa cells in human. In response to DNA damage induced by different chemotherapy drugs such as cisplatin and active in-vitro metabolite of cyclophosphamide 4 hydroperoxy cyclophophosphamide TAp63 is phosphorylated at Ser395 and Ser160/162 residues in human oocytes and granulosa cells without any increase in c-Abl expression. This finding in fact may explain why imatinib does not confer any protection against follicle loss induced by cisplatin. Another important point to emphasize that there might be some other mechanisms after chemotherapy exposure that contribute to depletion of follicle reserve in addition to direct toxic effects of chemotherapy drugs on the oocyte and granulosa cells. One of them is vascular damage that more commonly occurs after administration of particular chemotherapy drugs such as cyclophosphamide and cisplatin accelerates follicle loss and ageing process of the ovary^15,31^. The second mechanism is so-called burn out phenomenon. It was hypothesized that cyclophosphamide exposure activates phosphatidylinositol 3-kinase signaling pathway, which in turn causes premature activation of primordial follicles and hence “burn out” or early depletion of follicle pool^32^. It was also suggested as a third possible mechanism, at least in the mouse ovary that chemotherapy drugs may induce different mechanisms of follicle loss^33^. Taken together, these findings suggest that there could be a multitude of distinct pathogenetic mechanisms underlying chemotherapy-induced gonadotoxicity and ovarian failure.

In conclusion, imatinib mesylate itself may show cytotoxic effects on human ovary and does not appear to confer any protection against follicle loss induced by cisplatin in human ovary. Dissection of the molecular mechanisms underlying TAp63 triggered oocyte apoptosis is of paramount importance not only for a better understanding of pathologic forms of oocyte death but also for developing new pharmacological strategies to preserve ovarian reserve during cancer therapy in human ovary.

## Electronic supplementary material

Supplementary Figure 1

Supplementary Figure 2

Supplementary Figure 3

Supplementary Figure 4

Supplementary Material File

Supplementary Figure 5

Supplementary Figure 6

Supplementary Figure 7
