# Supplementary figures and images for: C-Abl is not actıvated in DNA damage-induced and Tap63-mediated oocyte apoptosıs in human ovary
Source: Cell Death Dis. 2018 Sep 20;9(10):943. doi: 10.1038/s41419-018-1026-7 (PMC6148240; doi:10.1038/s41419-018-1026-7)

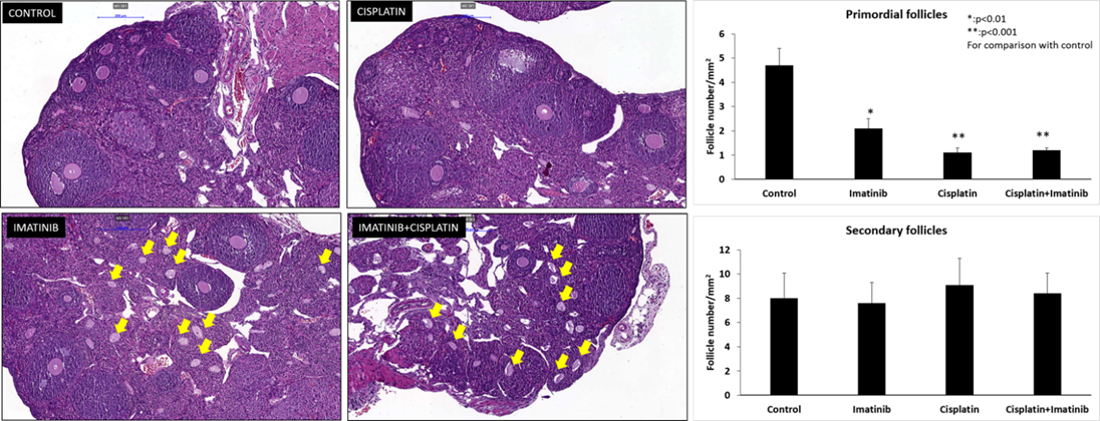

Supplement: Supplementary file 1 — Supplementary Figure 1 [file 41419_2018_1026_MOESM1_ESM.bmp]

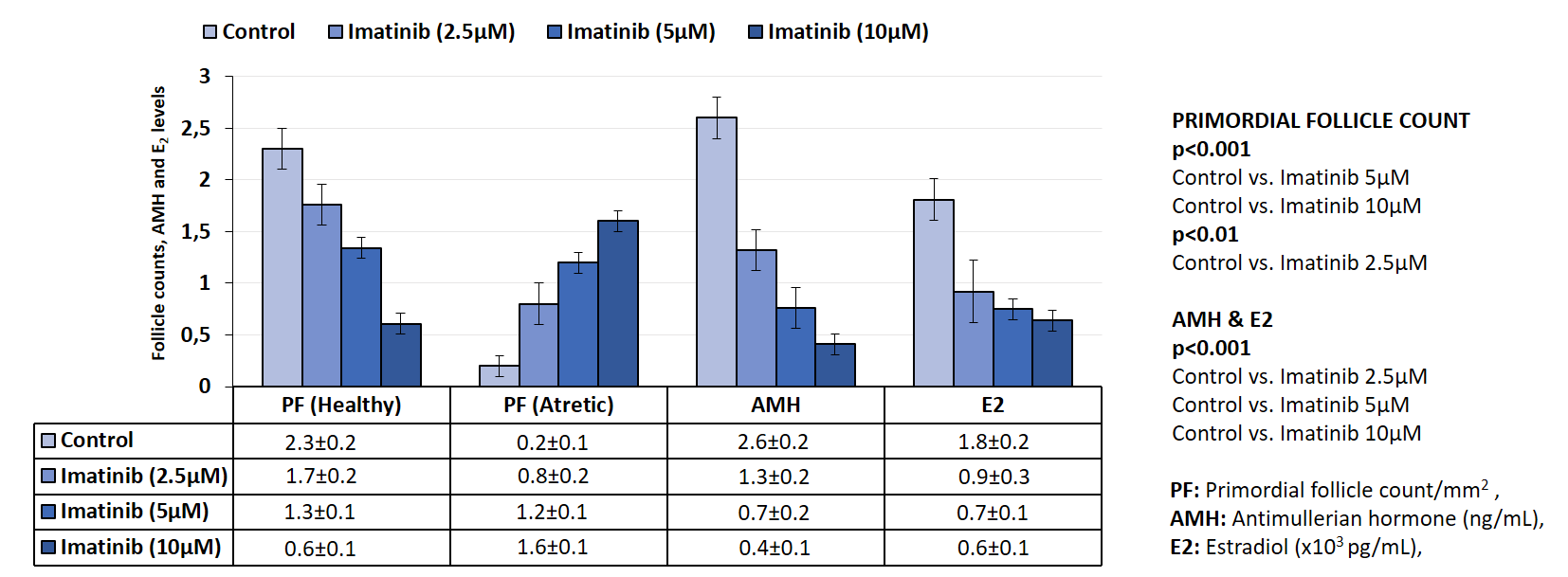

Supplement: Supplementary file 2 — Supplementary Figure 2 [file 41419_2018_1026_MOESM2_ESM.bmp]

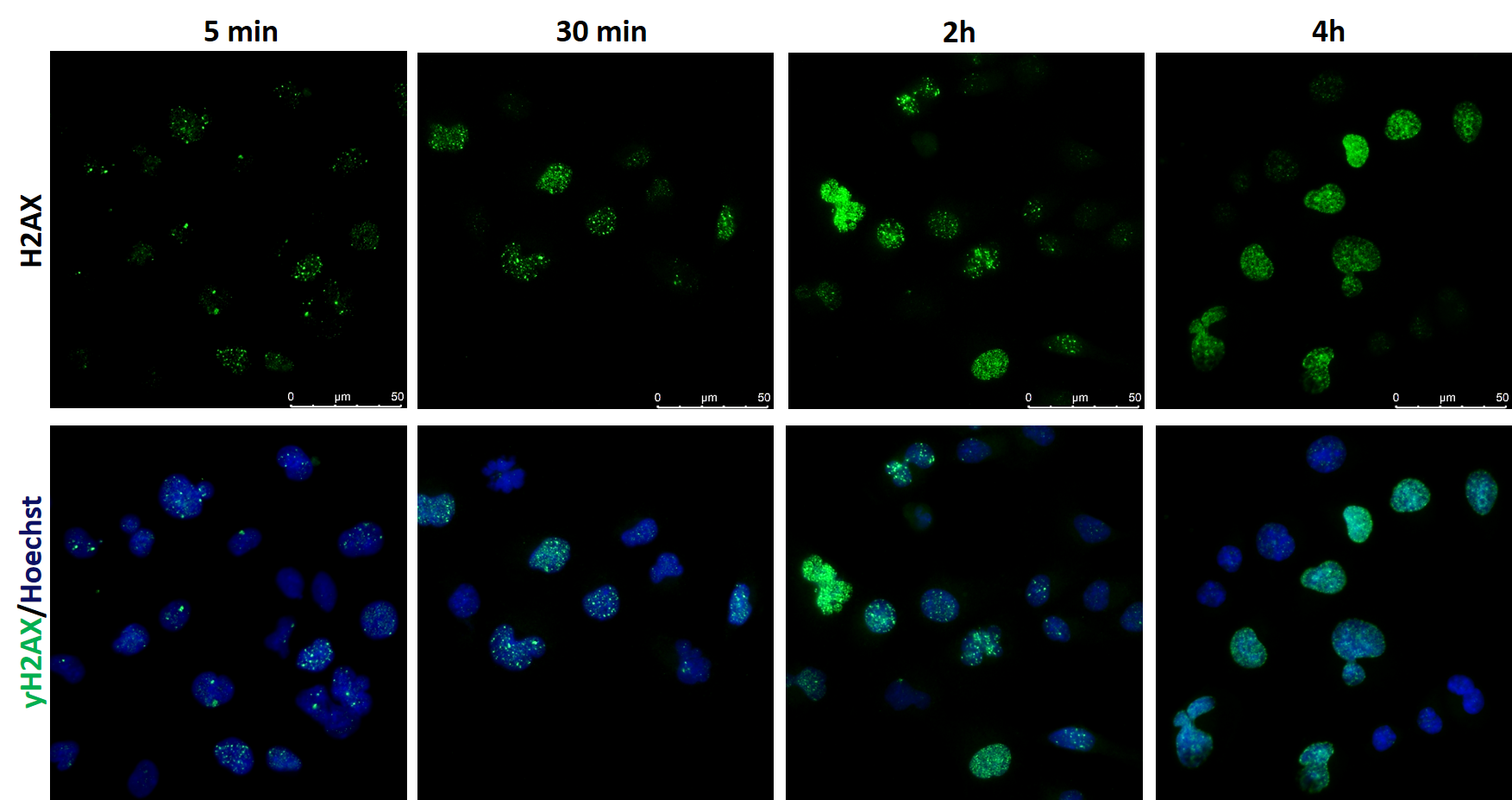

Supplement: Supplementary file 3 — Supplementary Figure 3 [file 41419_2018_1026_MOESM3_ESM.bmp]

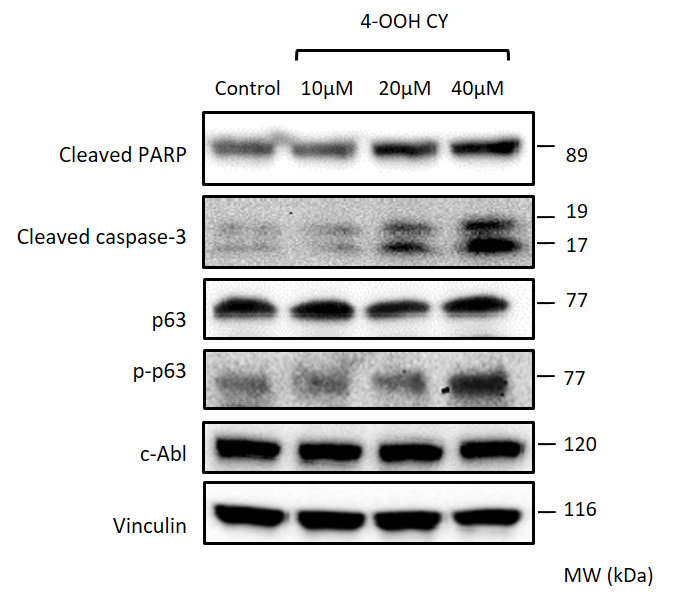

Supplement: Supplementary file 4 — Supplementary Figure 4 [file 41419_2018_1026_MOESM4_ESM.bmp]

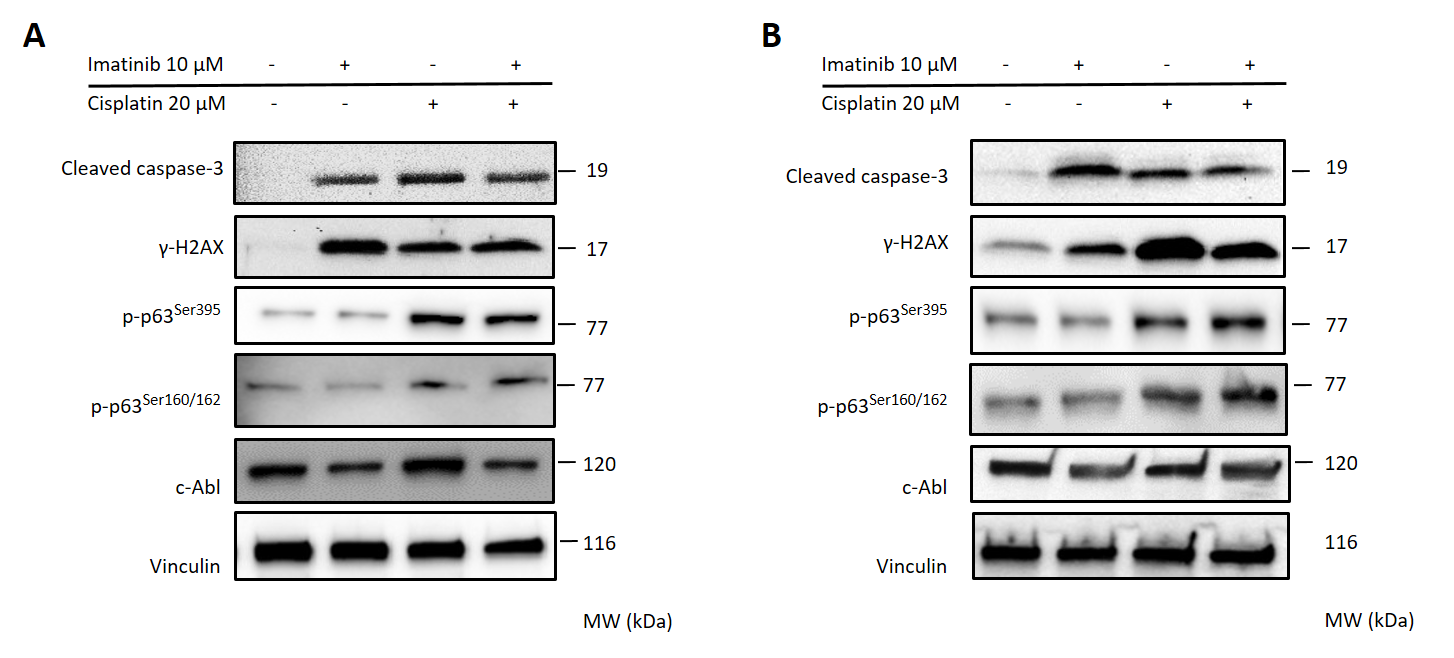

Supplement: Supplementary file 6 — Supplementary Figure 5 [file 41419_2018_1026_MOESM6_ESM.bmp]

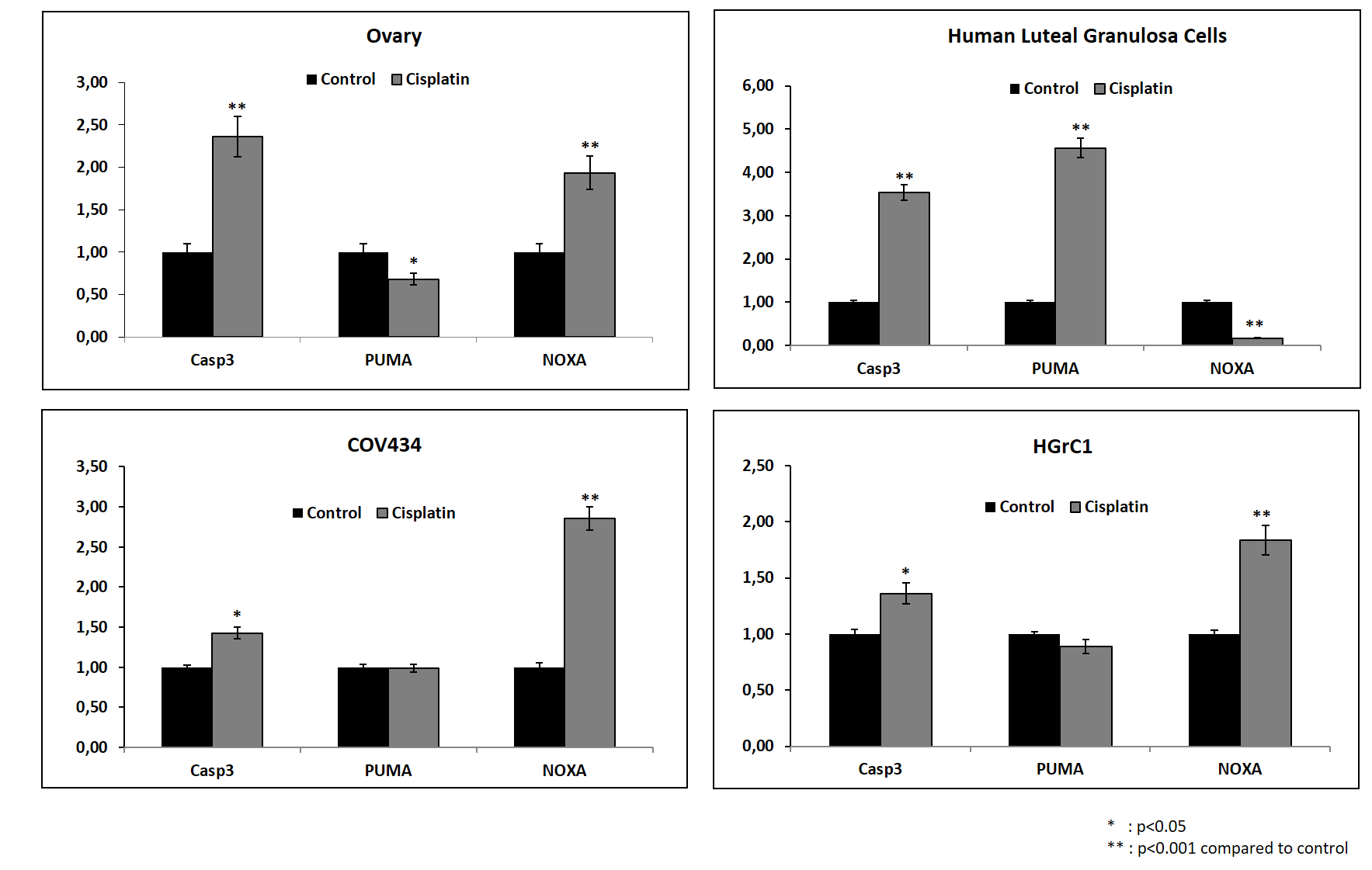

Supplement: Supplementary file 7 — Supplementary Figure 6 [file 41419_2018_1026_MOESM7_ESM.bmp]

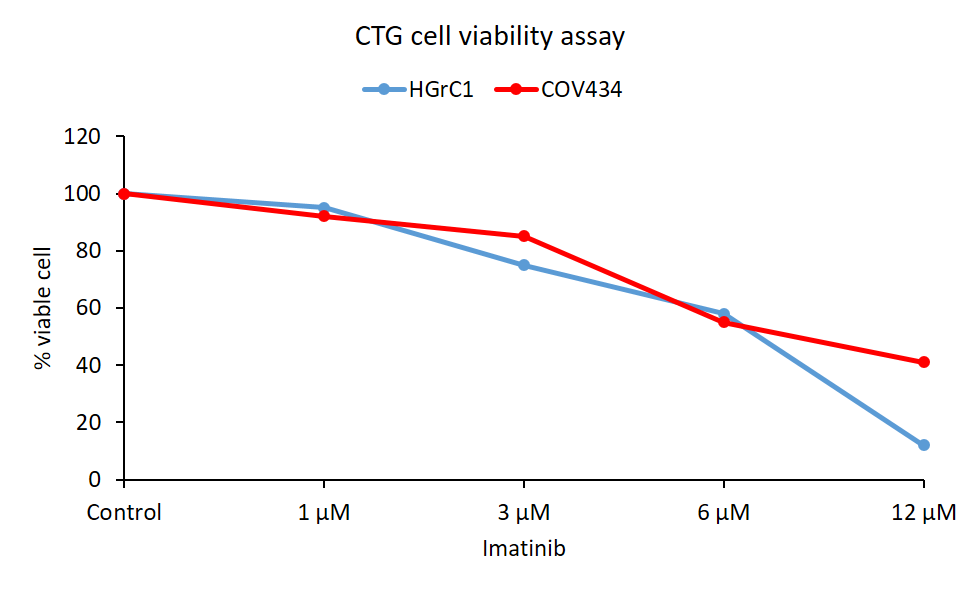

Supplement: Supplementary file 8 — Supplementary Figure 7 [file 41419_2018_1026_MOESM8_ESM.bmp]
