# Supplementary material for: C-Abl is not actıvated in DNA damage-induced and Tap63-mediated oocyte apoptosıs in human ovary
Source: Cell Death Dis. 2018 Sep 20;9(10):943. doi: 10.1038/s41419-018-1026-7 (PMC6148240; doi:10.1038/s41419-018-1026-7)
Supplement: Supplementary file 5 — Supplementary Material File [file 41419_2018_1026_MOESM5_ESM.docx]

**SUPPLEMENTARY FIGURES LEGENDS**

**Supplementary Figure-1: Histological examination of the animals’ own ovaries treated with cisplatin, imatinib or both.** The number of primordial follicles was significantly reduced in the animals treated with cisplatin. Imatinib treatment with cisplatin did not prevent cisplatin-induced follicle loss. Furthermore, the ovaries exposed to imatinib alone primordial follicle number was substantially reduced and there were multiple small follicles lacking oocytes (yellow arrows). The number of pre-antral and antral follicles (secondary follicles) were comparable among the control animals and those treated with cisplatin, imatinib or both.

**Supplementary Figure-2.** In-vitro treatment of human ovarian cortical pieces with imatinib at three different concentrations for 24h caused a dose-dependent decrease in primordial follicle number and their steroidogenic activity.

**Supplementary Figure-3:** In-vitro exposure of HGrC1 granulosa cells to cisplatin caused double strand DNA breaks as shown by increased expression of *γ*-H2AX ^Ser139^ as early as 5 min post-exposure. The intensity of the signal (the number of foci) was increased when duration of cisplatin exposure was extended up to 4h in immunofluorescence staining.

**Supplementary Figure-4:** Treatment of the granulosa cells with 4-hydroperoxy cyclophosphamide (4-OOH CY) at three different concentrations activated p63 pathway and apoptotic machinery as shown by increased expression of cleaved forms of PARP and caspase-3 without any notable change in the expression of c-Abl in immunoblotting.

**Supplementary Figure 5**: Treatment of COV434 (A) and human luteal granulosa cell (B) with cisplatin increased the expression of phosphorylated forms of γ-H2AX ^Ser139^, both p63^ser395^ and p63^ser160/162^ and cleaved caspase-3 without any increase in the expression of c-Abl. Imatinib treatment alone was associated with increased the expression of γ-H2AX phosphorylation ^Ser139^ and cleaved caspase-3 and decreased c-Abl expression without any increase in phosphorylated forms of p63 on both Ser395 and Ser160/162. Imatinib co-treatment with cisplatin did not cause any notable change in the expression of these markers in immunoblotting.

**Supplementary Figure-6:** RT-PCR profiler assay for gene expression of caspase-3, NOXA and PUMA before and after cisplatin exposure. While there is an almost 2-fold increase in NOXA expression in the ovarian cortical samples, and HGrC1 and COV434 cell lines exposed to cisplatin (20 μM) for 24 hours PUMA expression did not change. By contrast, in the luteal granulosa cells, PUMA was increased by 4.5-fold but NOXA was significantly down-regulated after the same duration of exposure to cisplatin. Asterix: for comparison to control

**Supplementary Figure-7:** Cell viability assay showed that imatinib caused a dose-dependent decrease in the viability of HGrC1 and COV434 granulosa cells.

**Supplementary Figure-8:** The expression of c-Abl was gradually diminished on immunoblotting when the HGrC1 granulosa cells were treated with increasing concentrations of imatinib or GNF-2, as a validation experiment showing in-vitro biological activity of these drugs.

**Supplementary Figure-1**

**
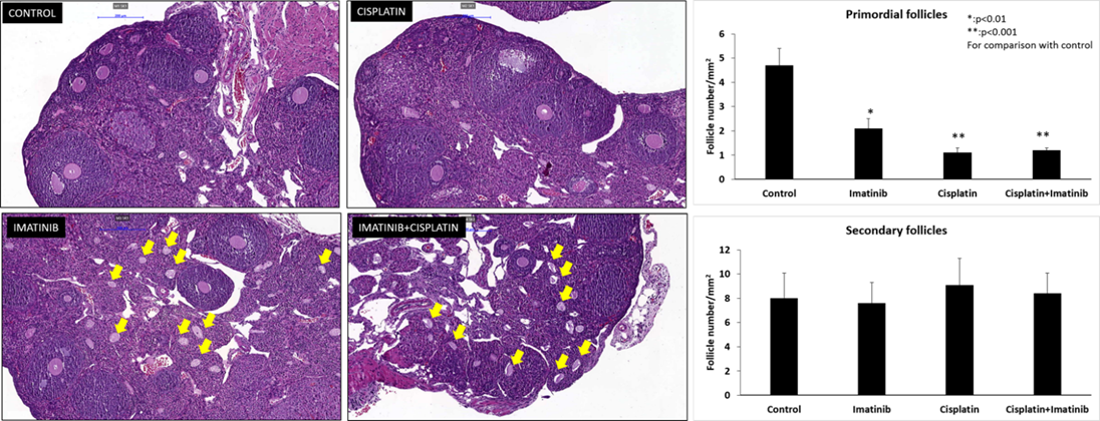
**

**Supplementary Figure-2.**


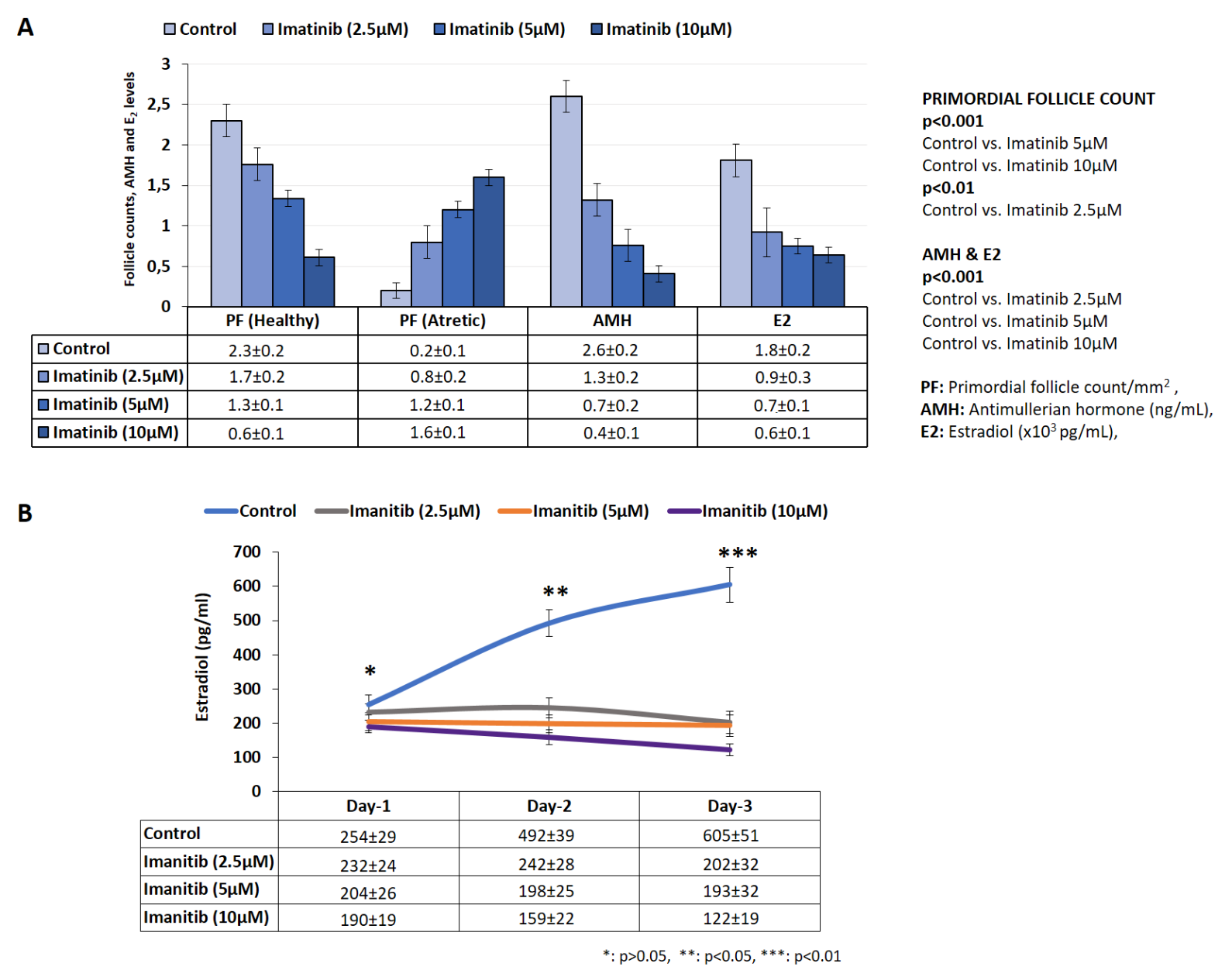


**Supplementary Figure-3.**

**
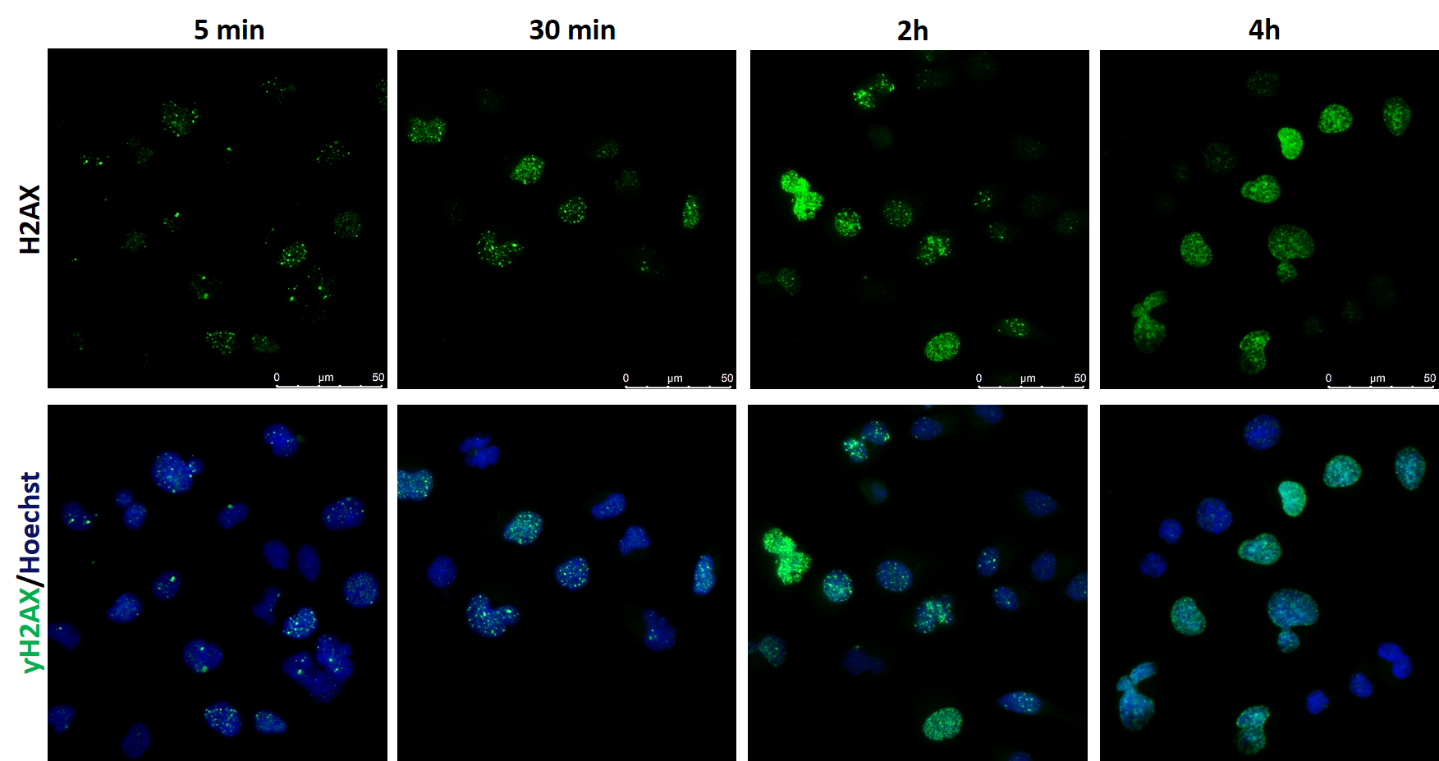
**

**Supplementary Figure-4.**

**
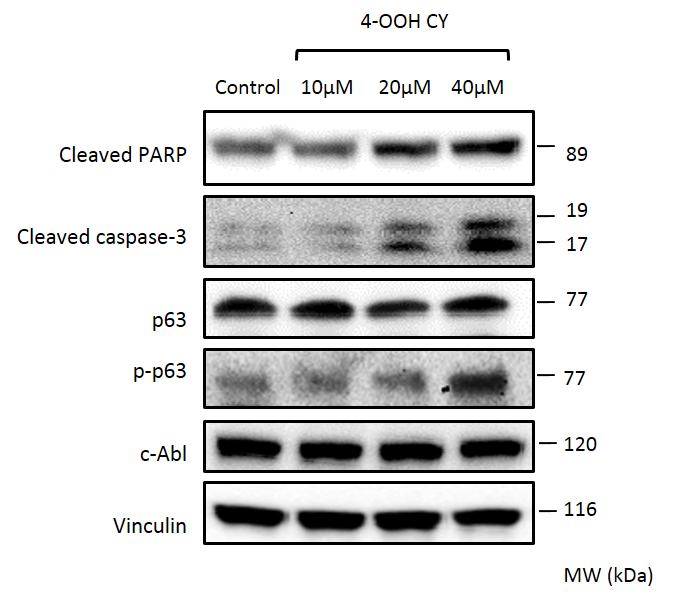
**

**Supplementary Figure-5**

**
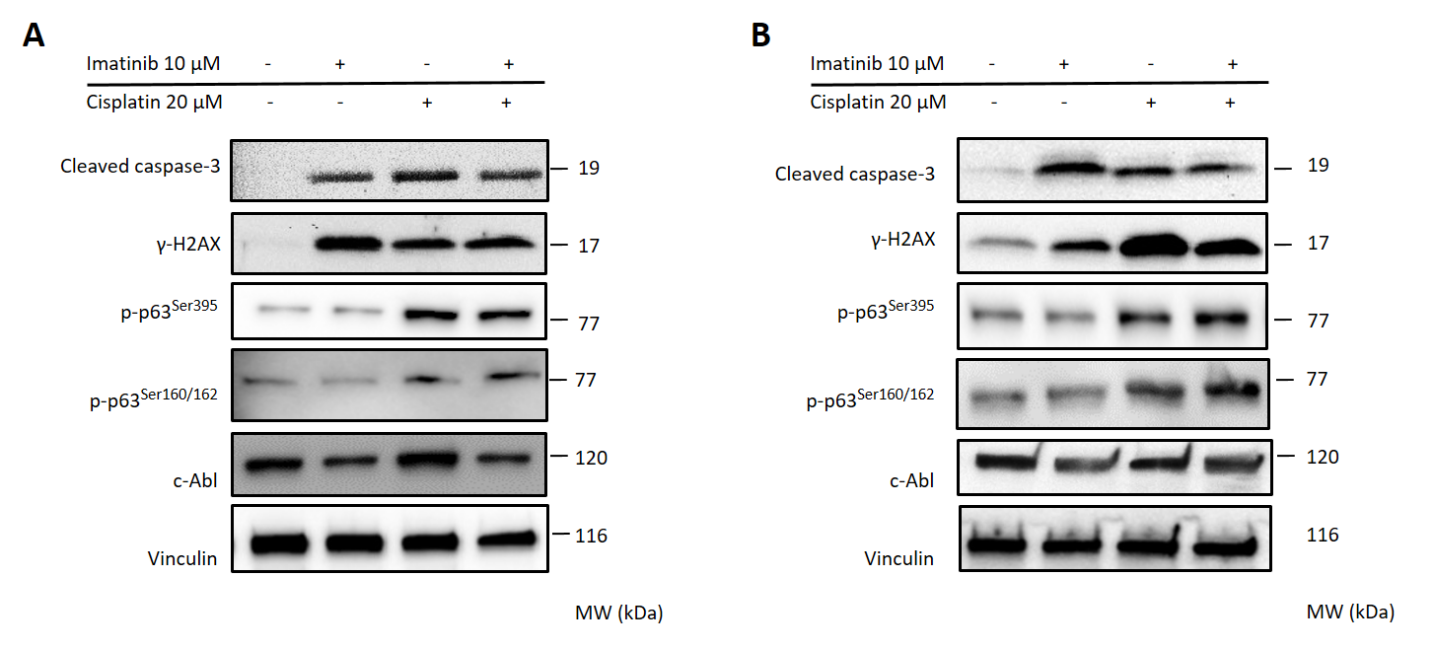
**

**Supplementary Figure-6**

**
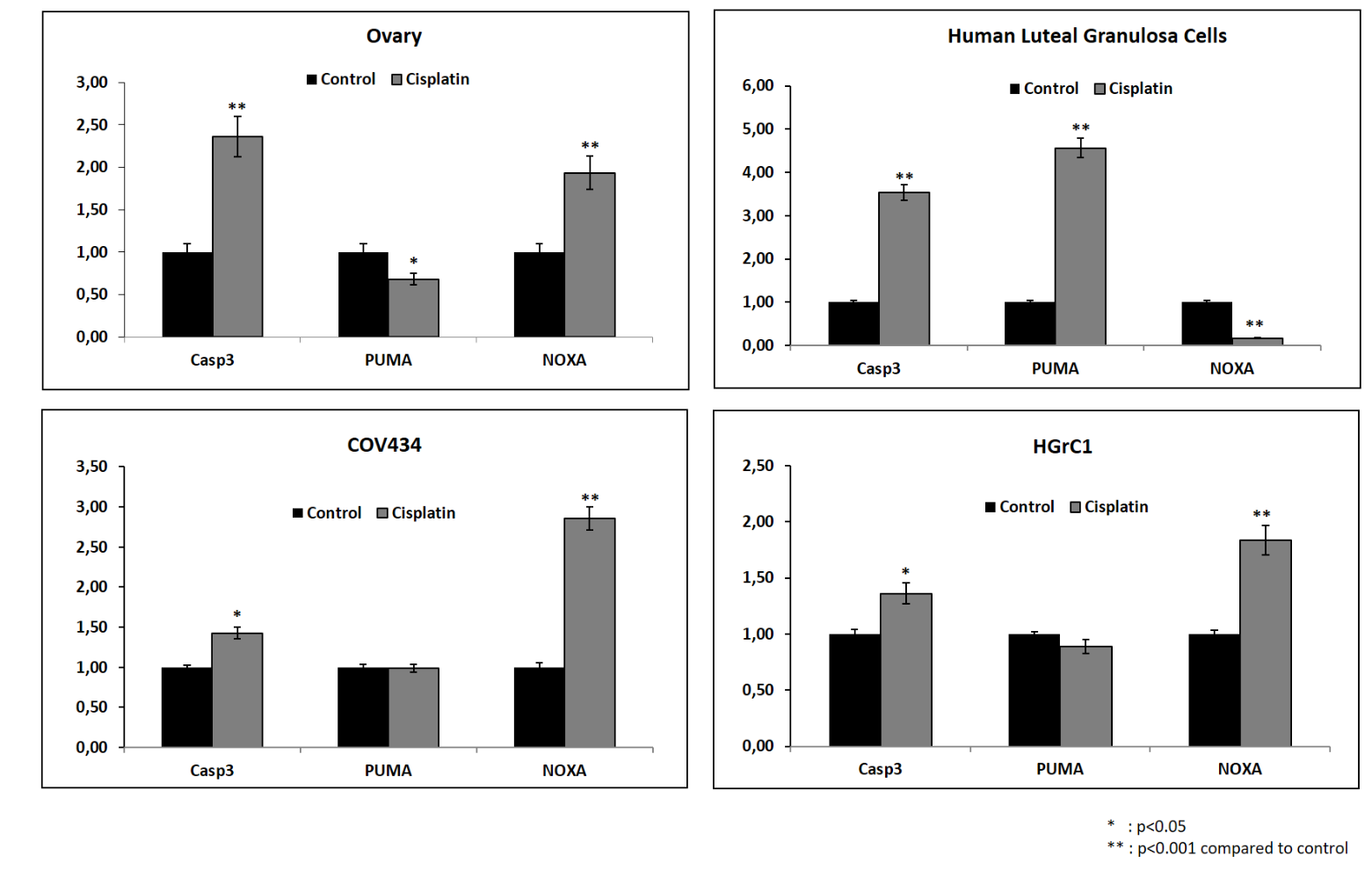
**

**Supplementary Figure-7**

**
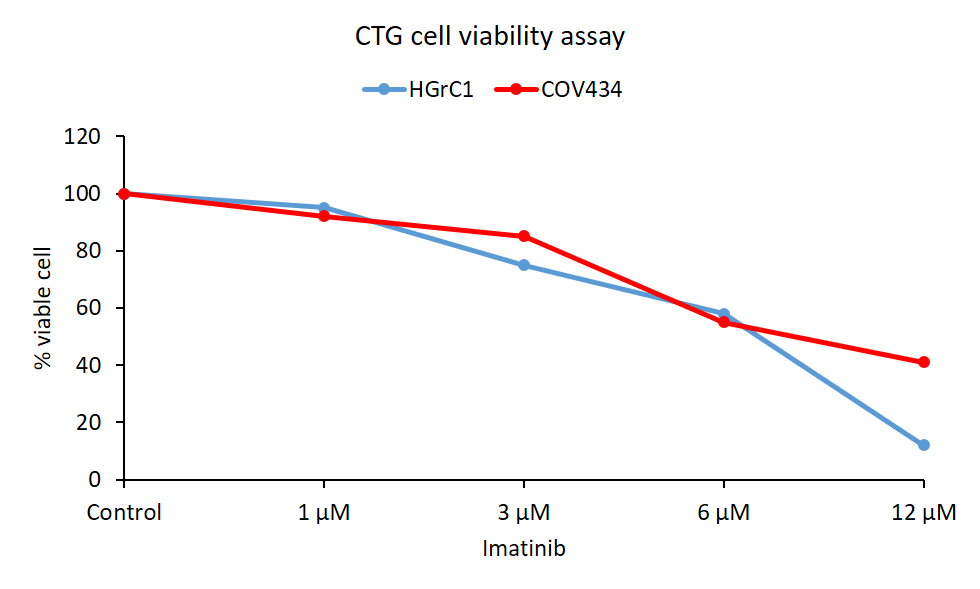
**

**
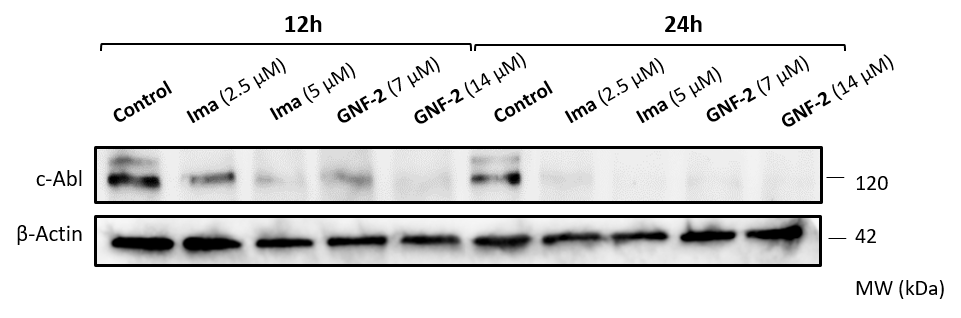
Supplementary Figure-8**
